# Dopamine in Sports: A Narrative Review on the Genetic and Epigenetic Factors Shaping Personality and Athletic Performance

**DOI:** 10.3390/ijms252111602

**Published:** 2024-10-29

**Authors:** Kinga Humińska-Lisowska

**Affiliations:** Faculty of Physical Education, Gdansk University of Physical Education and Sport, 80-336 Gdańsk, Poland; kinga.huminska-lisowska@awf.gda.pl; Tel.: +48-51-036-2693

**Keywords:** neurotransmitters, NEO-FFI, personality traits, polymorphism, athlete, mental resilience, Big Five model, SNP, VNTR

## Abstract

This narrative review examines the relationship between dopamine-related genetic polymorphisms, personality traits, and athletic success. Advances in sports genetics have identified specific single nucleotide polymorphisms (SNPs) in dopamine-related genes linked to personality traits crucial for athletic performance, such as motivation, cognitive function, and emotional resilience. This review clarifies how genetic variations can influence athletic predisposition through dopaminergic pathways and environmental interactions. Key findings reveal associations between specific SNPs and enhanced performance in various sports. For example, polymorphisms such as *COMT* Val158Met rs4680 and *BDNF* Val66Met rs6265 are associated with traits that could benefit performance, such as increased focus, stress resilience and conscientiousness, especially in martial arts. *DRD3* rs167771 is associated with higher agreeableness, benefiting teamwork in sports like football. This synthesis underscores the multidimensional role of genetics in shaping athletic ability and advocates for integrating genetic profiling into personalized training to optimize performance and well-being. However, research gaps remain, including the need for standardized training protocols and exploring gene–environment interactions in diverse populations. Future studies should focus on how genetic and epigenetic factors can inform tailored interventions to enhance both physical and psychological aspects of athletic performance. By bridging genetics, personality psychology, and exercise science, this review paves the way for innovative training and performance optimization strategies.

## 1. Introduction

Various factors influence success in sports, each contributing to an athlete’s competitive edge in varying degrees. An athlete’s physical conditioning, technical skills, mental resilience, and in-game tactical decisions are all critical components that determine the outcome of a contest. However, many experts—both theorists and practitioners—argue that the key to success in high-level competition lies in physical prowess and the athlete’s psychological attributes. Colloquially referred to as “winning with the mind”, this includes mental toughness, resilience, determination, and the ability to cope with setbacks [1,2].

Athletic success is a multifaceted phenomenon influenced by both genetic and environmental factors. Research suggests that approximately 66% of the variation in athletic status can be attributed to genetic factors [3]. These genetic influences comprise a range of traits, including histological, anthropometric, physiological, and psychological characteristics [4,5,6,7,8]. The remaining variation is influenced by environmental factors, such as deliberate practice, diet, and socioeconomic conditions [5,9,10]. This dynamic interplay highlights the importance of both inherent predispositions and external experiences in developing mental resilience and achieving athletic success.

As athletes continue to push the limits of human physical potential, and as elite competitors often exhibit near-maximal levels of physical preparation, it becomes clear that marginal physical differences cannot fully explain the performance gap at the highest levels. In this context, psychological predispositions, particularly personality traits influenced by dopamine pathways, may play a critical role in determining outcomes in competitive sports. Among the various neurotransmitter systems influencing psychological traits, dopamine stands out as a key regulator of motivation, reward processing, and cognitive functions, which are essential for athletic performance. Dopamine, a key neurotransmitter in the brain, is central to this discussion, as it regulates numerous physiological and psychological functions, including motivation, reward-seeking, learning, motor control, and executive functions [11,12]. Often referred to as the “pleasure molecule”, dopamine significantly influences not only daily mental processes, but also athletic performance and personality traits. It is critical for decision-making, stress resilience, and sustained performance in high-pressure athletic environments. Differences in dopamine-related genetic polymorphisms may explain the variance in mental resilience among athletes, offering insights into how they manage stress, motivation, and performance under pressure [13,14]. Therefore, understanding the specific aspects of dopamine pathways that influence personality traits relevant to athletic performance is fundamental to the advancement of sports science.

Biologically, personality and temperament are among the most complex and least understood aspects of human performance. The human mind is shaped by a dynamic interplay between biological structures, neurochemical processes, and environmental interactions [15,16]. Advances in research methods, including neuroimaging and psychometric testing, have provided greater insight into these psychological domains. For example, the NEO Five-Factor Inventory (NEO-FFI) has been widely used to assess key personality traits and their influence on behavior and performance. Studies using this tool suggest that traits such as conscientiousness and emotional stability (low neuroticism) are associated with greater resilience, self-discipline, and coping under competitive pressure, which are critical components of athletic success [17,18,19]. However, the genetic basis of these personality traits in athletes remains underexplored, indicating a significant gap in the literature.

Genetic factors such as polymorphisms in dopamine-related genes, including dopamine receptor genes (*DRD*), dopamine transporter gene (*DAT1*), and catechol-O-methyltransferase gene (*COMT*), have been extensively studied for their role in athletic performance and psychological traits, including impulsivity, stress tolerance, and goal-oriented persistence [13,20,21,22,23,24]. This review focuses on specific polymorphisms within these genes, such as *DRD2* Taq1A, *DRD3* rs167771, *DRD4* exon III VNTR, *COMT* Val158Met, *BDNF* Val66Met (rs6265), and *DAT1* 40 bp VNTR, and their influence on dopamine function and associated personality traits relevant to athletic performance. However, directly linking these genetic polymorphisms to personality traits and athletic success is a relatively new area of research that warrants further investigation.

In addition to genetic influences, epigenetic factors can be modified by external stimuli such as training intensity, stress exposure, and life experiences that affect dopamine pathways, adding further complexity to the relationship between biological processes and personality traits [25,26]. These epigenetic modifications can dynamically alter gene expression in response to environmental factors, such as physical activity, influencing an athlete’s psychological traits and performance over time. Recent research has shown that exercise-induced changes in DNA methylation can enhance dopamine synthesis and receptor sensitivity, potentially explaining some of the cognitive benefits observed in physically active individuals [27,28]. This review also explores how epigenetic modifications of dopamine-related genes contribute to personality traits that influence athletic performance, highlighting the importance of gene–environment interactions. Understanding how epigenetic modifications in dopamine-related genes affect personality traits such as motivation and stress resilience is essential for developing personalized training strategies.

The study of personality traits in athletes, particularly how these traits correlate with genetic and epigenetic factors, is a growing area of interest. Traits such as impulsivity, motivation, and resilience, partly regulated by dopamine pathways, have been shown to influence an athlete’s ability to perform under pressure and recover from setbacks. Despite these associations, comprehensive reviews have yet to coherently explore how dopamine-related genetic and epigenetic factors shape personality traits and athletic performance. To the best of the author’s knowledge, this is the first narrative review that integrates existing knowledge on genetic polymorphisms, personality traits, and athletic success, providing a unique synthesis of how these elements interact to influence athletic performance. By focusing specifically on dopamine-related genetic and epigenetic factors and their influence on personality traits that are critical for performance under pressure, this review aims to elucidate the complex interplay between genetics, personality, and athletic success.

Understanding the relationship between these traits and athletic success could lead to new strategies for improving training, recovery, and performance. Thus, this narrative review aims to fill this gap by exploring the complex interactions between dopamine pathways, genetic and epigenetic factors, and personality traits in athletes. By identifying specific dopamine-related genetic and epigenetic factors that affect key personality traits, this review aims to provide new insights that can inform personalized approaches to athlete development and performance optimization.

It is important to note that the majority of genetic studies examining personality traits in athletes have been conducted in European and North American populations. This geographic focus may limit the generalizability of findings to athletes from other regions and ethnic backgrounds. Genetic variations, including those in dopamine-related genes, may differ significantly between populations due to genetic diversity and evolutionary factors [29,30]. Expanding research to include more diverse populations is essential to gain a comprehensive understanding of how genetic and epigenetic factors influence personality traits and athletic performance worldwide.

Given the complexity of these interactions, this review explores the genetic and epigenetic factors that influence dopamine-related pathways and their specific impact on personality traits that are critical for athletic performance, such as motivation, stress resilience, and cognitive function. By synthesizing the latest research, this review aims to provide a comprehensive understanding of how these biological factors contribute to athletic success and psychological resilience, with implications for future sports science and mental health developments.

## 2. Personality Factors Influencing Athletic Success

An athlete’s effectiveness can be understood along several dimensions, including physical attributes (motor skills—encompassing a wide range of physiological and biomechanical factors, including strength, speed, endurance, agility, coordination, and flexibility) [31], cognitive decision-making factors [32], and personality and temperamental traits [33,34]. While physical skills are essential, especially in sports requiring strength, speed, and endurance, there is increasing reliance on cognitive skills and personality traits in high-pressure competitive environments. So far, however, there has been little discussion about the genetic and environmental contributions to these personality traits and how they specifically influence athletic success. Research has shown that between 70% and 85% of successful and unsuccessful athletes can be distinguished using general psychological measures of personality structure and mood state [35]. These findings underscore the significant role of personality type in mediating athletic success, suggesting that psychological traits are vital performance components. However, the genetic basis of these emotional and mental traits, which may predispose athletes to outstanding performance, remains underexplored. This calls for a deeper examination of how genetics and environmental factors contribute to these personality dimensions in athletes.

### 2.1. The Influence of Big Five Personality Traits on Athletic Performance

Personality traits, as defined by the Big Five model, include openness, conscientiousness, extraversion, agreeableness, and neuroticism and have been shown to influence an athlete’s motivation, behavior, and overall response to training [36]. These five dimensions capture important differences in how individuals experience emotions, approach motivation, and engage in cognitive processes [37]. The NEO Five-Factor Inventory (NEO-FFI) [38] is commonly used to assess these traits. It provides insight into how they may predict an athlete’s engagement in training, interaction with teammates, and handling of competitive pressure. Furthermore, genetic factors can account for approximately 50–60% of the variance in Big Five personality traits [39], highlighting the role of innate predispositions in shaping athletic behavior. For example, a twin study from Germany and Serbia found that 63–79% of the variance in perceived parenting dimensions, such as support and organization, could be attributed to genetic influences [40]. This suggests that an athlete’s personality is shaped by a dynamic interplay between genetic and environmental factors that may contribute to their potential for success.

Despite extensive research on personality and athletic performance, the nuances of how each trait affects different sports and activities remain underexplored. One possible explanation may reside in the differing physical demands of different sports, which may interact uniquely with individual personality profiles. In this context, mental toughness, drive, and the ability to cope with stress are critical personality traits that, arguably, contribute to athletic success and result from a combination of genetic predispositions and environmental experiences. These traits are deeply rooted in neurobiological mechanisms, and understanding their origins can provide valuable insights into the psychological aspects of athletic performance.

Studies suggest that conscientiousness, emotional stability (low neuroticism), and extraversion are important predictors of athletic performance, with conscientiousness being particularly influential. Characterized by self-discipline, organization, and a strong focus on goals, conscientiousness has consistently been associated with tremendous athletic success. Athletes who score high on conscientiousness tend to be more disciplined in their training routines and adhere to long-term goals, directly contributing to improved performance [17]. This trait is essential for self-regulation and persistence, making it particularly relevant in individual sports, where personal motivation and discipline are critical factors for success [41,42]. Conscientiousness is also associated with greater consistency in training commitment, which may lead to better physical and psychological outcomes in both short- and long-term athletic development. In twin studies, conscientiousness was found to have a heritability estimate of approximately 44%, thereby implying a significant genetic influence in addition to environmental factors (e.g., training and coaching) [43].

Recent research has further emphasized the nuanced role of conscientiousness in a team-sport environment. According to the recent review by Shuai et al. [44], conscientiousness shows a positive association with athletic performance in team sports such as soccer and basketball [45,46]. Traits associated with conscientiousness, such as self-discipline, diligence, responsibility, and reliability, are integral to enhancing performance in team settings, where individual efforts directly influence collective outcomes. However, in contrast to its role in team sports, conscientiousness does not significantly influence performance in individual endurance sports, such as swimming and aerobic activities. This is consistent with previous research suggesting that conscientiousness and motivation may not predict performance in endurance sports [47]. Additionally, a meta-analysis of sixty-four studies, including 88,400 participants, has shown that while conscientiousness is generally positively correlated with physical activity, the strength and significance of this relationship can vary due to methodological and sample characteristics [48]. Wilson and Dishman found that factors such as the quality of physical activity measurement, study design, and participant demographics can moderate this association [48]. One possible explanation could be that conscientiousness influences physical activity differently in different contexts, sports types, or age groups.

Emotional stability, often referred to as low neuroticism, is another essential trait linked to athletic performance. Athletes who demonstrate emotional stability can better manage stress and cope with the pressure of competition. This trait is critical for maintaining focus and delivering consistent performance, especially in high-stakes environments such as professional sports leagues or the Olympics [49], soccer [50], boxing, and martial arts. Conversely, high levels of neuroticism, which is associated with emotional instability and anxiety, can hinder athletic performance by increasing stress, decreasing confidence, and decreasing focus during competition [51]. Thus, athletes with higher emotional stability are generally more resilient in facing challenges and setbacks, which is a critical factor for success in sports [52]. The heritability of neuroticism has been estimated at 41%, suggesting a strong genetic basis, although environmental factors play an important role in modulating this trait [43]. Consistent with these observations, both Rhodes and Smith [53] and Wilson and Dishman [48] reported significant negative associations between neuroticism and physical activity levels. Individuals lower in neuroticism tended to engage in physical activity more frequently and intensely. This supports the notion that emotional stability promotes an active lifestyle, potentially improving engagement in long-term training among athletes.

Consistent with genetic influences on personality and physical activity, Butković et al. conducted a twin study involving 339 twin pairs (105 MZ and 234 DZ) to estimate the heritability of exercise behavior by examining the genetic and environmental contributions of associations between personality traits and physical activity [54]. They found that heritability estimates for physical activity variables ranged from 55% to 69%, indicating a substantial genetic influence on exercising behaviors. In addition, significant phenotypic associations were found between physical activity and the personality traits of neuroticism and extraversion. Specifically, individuals higher in extraversion and lower in neuroticism reported exercising more frequently and more intensely. Notably, the study showed that the phenotypic associations between personality traits and physical activity were due to overlapping genetic influences. This implies that the genetic factors responsible for traits like extraversion and neuroticism also play a role in influencing exercise behavior. These findings support the “nature” hypothesis, which suggests the existence of biological differences in predisposition to regular physical activity [54]. However, contrary to expectations, the study did not find significant correlations between conscientiousness and physical activity variables. This challenges the assumption that conscientiousness is a universal predictor of exercise behavior and suggests that its role may be more complex. A possible explanation may be that conscientiousness influences physical activity differently depending on age, type of sport, or cultural context.

Extraversion, characterized by sociability, assertiveness, and enthusiasm, also plays a significant role in athletic performance, particularly in team sports. Extraverted athletes often excel in environments where frequent interactions with teammates, coaches, and competitors foster effective communication and collaboration, creating a positive atmosphere that enhances performance [55]. This advantage is especially pronounced in team-based sports, such as basketball and football, where camaraderie and mutual support are crucial for success. For example, NBA players with high levels of extraversion have been found to perform better across various measures, including an enhanced ability to tolerate physical pain, which is essential in team sports where physical and mental endurance are required [46]. Extraversion has also been linked to higher levels of motivation and engagement during training, which can translate into better performance outcomes, especially in sports that strongly rely on teamwork and group dynamics [56,57]. Moreover, extraversion has been linked to managing stress more effectively in team settings, such as football, where a task-oriented approach, enabled by extraverted traits, can improve an athlete’s ability to handle competitive pressure [45]. Interestingly, individual event champions, including those in individual sports, have reported high levels of extraversion, suggesting that this trait benefits athletes beyond team sports [58]. Extraverted athletes are more likely to seek out social support and use it as a coping mechanism, further enhancing their ability to manage stress during competition. Additionally, extraversion has been associated with improved aerobic capacity, reflecting the energetic and fast-paced lifestyles often seen in extraverted athletes [59]. The combination of these traits allows extraverted athletes to excel by capitalizing on teamwork and maintaining high levels of personal energy and motivation. Extraversion has a heritability estimate of 53%, indicating substantial genetic influence, although environmental factors again shape this feature [43]. Meta-analyses by Rhodes and Smith (2006) and Wilson and Dishman (2015) found significant positive associations between extraversion and physical activity levels [48,53]. However, the magnitude of these associations varies, and factors such as geographic region, gender, and the definition of physical activity may moderate this relationship. Taken together, it appears that the influence of extraversion on athletic performance is more nuanced than previously thought. Supporting the genetic basis of this relationship, Butković et al. found that the phenotypic association between extraversion and physical activity was primarily due to shared genetic factors [54]. That is, genetic influences accounted for a significant portion of the correlation between extraversion and physical activity behavior. This highlights the role of genetics in understanding how personality traits influence exercise behavior, while suggesting that individuals high in extraversion may be genetically predisposed to have a physically active lifestyle.

The willingness to embrace new ideas and creativity, known as openness, also contributes to athletic performance. Athletes high in openness are often more adaptable to new strategies and training methods, making them well suited to sports that require creative problem-solving and tactical flexibility [60]. This trait is particularly beneficial in dynamic sports environments where athletes must quickly adapt to changing circumstances. Research has shown that athletes with high openness to experience tend to be more curious, imaginative, and willing to try new approaches, which can lead to improved learning and skills [61]. In addition, athletes who score high on openness often seek out diverse experiences and exhibit curiosity about their surroundings, consistent with the mental and physical challenges of high-risk and technical sports such as rock climbing [62]. For example, both sport climbers and boulderers have shown a positive correlation between openness scores and climbing performance. Openness has the highest heritability estimate of the Big Five, at 61%, suggesting a strong genetic basis for this trait [43]. Despite these potential benefits, Wilson and Dishman found that openness had only a small positive association with physical activity (r = 0.0344), suggesting that this trait may not strongly influence general exercising behaviors [48]. It is possible that openness affects other aspects of exercise performance that are not directly measured by exercise frequency or intensity. Alternatively, openness could be a more influential trait in a specific sporting context, as opposed to the general expression of physical activity.

Risk-taking, a key subcomponent of openness, is particularly prevalent among athletes who participate in high-risk sports. This trait fosters a willingness to engage in novel and uncertain situations, contributing to improved performance in disciplines that require experiential openness and quick decision-making [63]. For example, athletes in high-risk sports, such as rock climbing, skydiving, and martial arts, often exhibit higher levels of openness due to the demands of their sport. Similarly, our previous study observed higher openness, extraversion, and conscientiousness in martial arts compared to controls [64]. Higher openness has also been considered necessary in American football, where openness to new strategies and tactical shifts is essential for adapting to the evolving nature of the game. Some researchers advocate systematic personality testing, particularly for openness, during the selection process for elite American football players to ensure they can handle the demands of elite competition [65]. This suggests that openness may contribute to an athlete’s ability to innovate and adapt in complex, fast-paced sporting environments.

Agreeableness, characterized by cooperativeness, trust, and compassion, is essential for fostering positive relationships in team sports. Athletes with higher agreeableness tend to resolve conflicts and maintain team cohesion, which improves overall performance. In contrast, athletes with low levels of agreeableness, extraversion, and/or emotional stability tend to exhibit aggressive behaviors [17]. Team-sport athletes generally score higher on agreeableness than those in individual sports, due to the social demands of teamwork [66]. However, extremely high levels of agreeableness may hinder performance in competitive contexts, as seen in basketball, beach volleyball, and American football, where assertiveness and individual drive are crucial [46,67]. In contrast, individual sports such as rowing and canoeing show a positive relationship between agreeableness and performance. Elite rowers with higher agreeableness tend to perform better, probably because achieving a balance between teamwork and individual focus is a necessary requirement for this sport [68]. The heritability estimate for agreeableness is approximately 41%, suggesting a combination of genetic and environmental contributions [43]. Interestingly, both Rhodes and Smith [53] and Wilson and Dishman [48] found no significant associations between agreeableness and physical activity levels. This highlights the complexity of the relationship between personality traits and various aspects of athletic performance, suggesting that agreeableness may not directly influence exercise behavior, but may still play a role in team dynamics and cohesion.

A study by Allen et al. examined the effects of the Big Five personality dimensions on coping strategies in sports among 253 athletes [55]. Those athletes who were extraverted, emotionally stable, and open to new experiences were more likely to use problem-focused coping strategies, which are critical for managing the demands of competition. In addition, conscientious athletes and those high in extraversion, openness, and agreeableness tended to use emotion-focused coping strategies. In contrast, athletes low in openness or high in neuroticism tended to use avoidance coping strategies. This suggests that personality profiles may help predict coping strategies and levels of athletic engagement, which are critical for success in individual and team sports. Another study by Malinauskas et al. examined the relationship between personality traits and athletic performance in 376 young men, including 169 athletes and 207 non-athletes [69]. The study found that athletes scored higher on conscientiousness, and team-sport athletes scored higher on extraversion than endurance athletes, highlighting the importance of personality traits in different sporting contexts. These trends indicate that certain traits are beneficial, depending on the specific demands of the sport. However, findings regarding conscientiousness are not always consistent. As mentioned above, Wilson and Dishman found that the relationship between conscientiousness and physical activity may be moderated by various factors [48]. One possible explanation for this discrepancy could be differences in the measurement of physical activity or the age range of participants, as younger individuals may exhibit different behavioral patterns.

Butković et al. also found that the phenotypic associations between personality traits and physical activity were due to overlapping genetic influences [54]. This suggests that genetic factors contribute to personality and physical activity behavior, highlighting the importance of considering genetic and environmental factors when investigating how personality traits influence physical activity. The high level of heritability in describing physical activity indicates that individuals might have a genetic inclination to exercise regularly, which could impact their potential when transitioning towards competitive sport and increased training demands.

The general profile of athletes, in terms of the Big Five personality traits, typically shows lower levels of neuroticism and higher levels of extraversion and conscientiousness [33,50,70,71,72,73,74]. A study conducted by Piepiora et al. on 1260 Polish male athletes, including 118 champions (athletes with significant competitive achievements), found that champions were characterized by lower levels of neuroticism and higher levels of extraversion, openness to experience, agreeableness, and conscientiousness compared to other athletes [52]. Notably, low neuroticism emerged as a critical determinant of being classified as a champion. However, it is important to note that personality profiles can vary depending on the specific sport and its demands, making it challenging to define a universally favorable personality type for athletic success. This equivocality is reinforced by the profiling of 119 athletes aged 19–34, including 100 Polish professional athletes (30 basketball players, 40 football players, 30 Kyokushin karate competitors) and 19 elite athletes (including 3 Olympic medalists) [75]. The NEO-FFI Personality Questionnaire study revealed noteworthy variances in neuroticism, extraversion, agreeableness, and conscientiousness among the different groups. Notably, low neuroticism emerged as a critical determinant of being classified as a champion, suggesting that this trait contributes significantly to athletic success. However, no direct correlation was found between personality traits and the likelihood of becoming a professional athlete, suggesting that other factors may influence progression to the elite level.

Interestingly, research on competitive athletes participating in eSports [76], such as the video game League of Legends, found that higher-ranked players tended to be less extroverted and agreeable, but at the same time were more open to new experiences. This evidence highlights how personality traits can affect success differently depending on the context (e.g., physical versus non-physical). Collectively, these findings underscore the variability in how personality traits manifest across different domains of competitive activity, from traditional sports to eSports, and further illustrate the complex relationship between personality and performance.

Conscientiousness, extraversion, and emotional stability are potent predictors of an athlete’s commitment to training programs, ability to cope with failure, and overall performance. For example, extraversion enhances team dynamics and cooperation, which is particularly beneficial in team sports [56,57]. Conversely, high levels of neuroticism, which are associated with emotional instability and stress, can hinder performance by increasing anxiety and decreasing focus during competition [77,78]. Athletes with a high openness to experience tend to be more adaptable, open to new training methods, and thus capable of enhancing their overall skills and performance [79]. While less often emphasized, agreeableness contributes to better teamwork and communication, which is essential for success in team settings. High conscientiousness, associated with discipline and goal orientation, is particularly important in individual sports, where self-regulation and personal motivation are key [80,81,82].

Despite these findings, the role of personality traits in predicting athletic success remains complex and multifaceted. It may vary across sports, levels of competition, and cultural contexts. The variability in research findings highlights the need for more nuanced studies that consider factors such as sport type, level of competition, and cultural background, to better understand how personality traits interact with specific sporting environments.

In summary, personality traits, as assessed by the Big Five model, provide valuable insights into how athletes approach training, competition, and team dynamics. These traits influence individual motivation, coping strategies, and overall performance and play a critical role in determining athletic success in various sporting contexts. Recognizing the importance of these personality factors can help coaches, trainers, and athletes develop more effective training programs and strategies that align with individual psychological profiles.

### 2.2. The Interaction Between Personality and Cognitive Traits in Athletic Performance

While cognitive and personality traits are often studied separately, their interaction is essential to understanding the psychological makeup of elite athletes. Cognitive flexibility, the ability to adapt to new and changing situations, is often associated with personality traits such as openness to experience and emotional stability [83]. Athletes who demonstrate cognitive flexibility and emotional resilience are better equipped to excel in complex, dynamic sports environments where adaptability and quick decision-making are critical to success [84].

Openness to experience has been associated with greater cognitive flexibility, enabling athletes to adopt new strategies, adapt to unexpected situations, adapt to unexpected situations, and think creatively under pressure [85]. This is particularly valuable in sports such as soccer, where athletes must respond quickly to changing game conditions. Emotional stability, or low neuroticism, also plays a critical role in helping athletes maintain focus and make rational decisions during high-stress moments, reducing the negative impact of anxiety on performance [86,87].

However, there has been limited discussion about how the interaction of these personality traits and cognitive functions directly influences particular athletic results. A possible explanation for this is the complexity of measuring cognitive flexibility in real-world sports settings and the variability of individual sports compared to team sports. Research suggests that athletes who combine high cognitive flexibility with emotional stability tend to outperform their peers in dynamic sports. In soccer, for example, players who exhibit cognitive flexibility are more adept at switching between offensive and defensive strategies, adapting to the movements of their teammates and opponents in real time. These athletes are more likely to anticipate and respond to changes in the game’s flow, giving them a strategic advantage [84].

In addition, working memory capacity, the ability to retain and manipulate information, is closely tied to cognitive flexibility and has been linked to conscientiousness. Athletes with higher conscientiousness and working strong memory are more likely to succeed in sports requiring long-term planning and sustained attention, as seen in endurance sports like marathon running, where strategic thinking and self-discipline are essential [83]. These athletes are more likely to stick to training programs, follow tactical plans, and consistently execute strategies under pressure, demonstrating how the interaction between personality and cognitive traits can predict long-term success.

Despite these findings, more research is needed to explore the interaction between personality and cognitive traits in different types of sports. To date, studies have predominantly focused on team sports like soccer, leaving individual sports as a less explored area. One possible explanation for this disparity is that team sports inherently require more dynamic interactions and rapid decision-making, so cognitive flexibility and personality interactions become natural targets for assessment.

The synergy between cognitive and personality traits can shape how athletes respond to physical challenges and might influence their ability to develop and maintain competitive strategies over time. As sports science increasingly integrates psychological measurements into applied research, understanding these interactions offers new avenues for enhancing athletes’ performance and mental well-being.

### 2.3. Biological Basis of Personality and Cognitive Factors in Athletes

Recent advances in neuroscience and genetics have shed light on the biological underpinnings of personality and cognitive traits, providing a deeper understanding of their role in athletic performance. Despite this progress, there remains a gap in research specifically examining how these biological mechanisms influence athlete development and/or competitive abilities. For example, dopaminergic pathways in the brain are heavily involved in regulating motivation, reward processing, and executive functions, which are critical for cognitive performance and personality traits such as impulsivity and resilience [13].

Dopamine, often referred to as the “pleasure neurotransmitter” or “the hormone of motivation to act and search for new emotions” [88], is a crucial chemical messenger in the brain, playing a key role in regulating a wide array of functions, including behavior, motivation, learning, movement control, executive functions, and emotional responses [11,12]. Discovered by Arvid Carlsson in the late 1950s [89], dopamine’s involvement in the brain’s reward system has been extensively studied, particularly in contexts that involve reinforcement, learning, “risky decisions”, and the pursuit of pleasure [90,91]. Variations in dopaminergic activity may contribute to individual differences in athletic performance by affecting these functions.

Dopamine neurons, primarily located in the ventral tegmental area (VTA) and substantia nigra (SN) of the brain, also present in the retrorubral field (RRF) (Menegas et al., 2015), play a crucial role in regulating various behaviors, including reward-seeking, motivation, and cognitive functions [92]. These neurons project to several brain regions, including the prefrontal cortex and striatum, forming pathways essential for dopamine’s wide-ranging effects [93]. Understanding these pathways is essential to understanding how genetic and neurobiological factors might enhance athletic success.

The actions of dopamine are mediated by five receptor subtypes—D1, D2, D3, D4, and D5—all of which belong to the family of G-protein-coupled receptors (GPCRs), which modulate intracellular pathways [94]. These receptors play a central role in the central nervous system, influencing everything from movement [95] and cognition [96,97] to emotional responses [11,98,99]. Dopamine synthesis begins with the amino acids tyrosine and phenylalanine, commonly found in protein-rich foods [100]. Anticipation of reward triggers this synthesis, resulting in a “feel good” sensation that motivates behavior. This dopaminergic modulation of motivation and reward could explain why some individuals are more driven to engage in rigorous training and competitive sport.

However, there has been little discussion about the specific genetic variations in dopaminergic pathways that affect athletic performance. Studying these genetic factors could provide valuable insights into the biological basis of motivation and resilience in athletes. Understanding these mechanisms may lead to more effective training strategies that take advantage of individual neurobiological profiles.

## 3. Genetic and Epigenetic Influences on Dopamine in Athletic Performance

### 3.1. Genetic Factors Influencing Dopamine Function and Athletic Behavior

Genetic and epigenetic factors strongly influence dopamine production and functional regulation [101]. Several genes regulate dopaminergic neurotransmission, and polymorphisms in these genes have significant functional and behavioral consequences [24,102,103]. Essential genes include those associated with dopamine receptor density, such as dopamine receptor genes (*DRD*), as well as those involved in dopamine metabolism, including the catechol-O-methyltransferase (*COMT*) gene [104,105,106] or dopamine transporter gene (*DAT1*) [107,108]. Variations in these genes can affect dopamine-related behaviors by altering receptor density, neurotransmitter metabolism, and dopamine reuptake.

Furthermore, it is important to emphasize that athletic performance is a highly polygenic trait, meaning it is influenced by numerous genes, each contributing a small effect. An athlete’s polygenic profile can significantly influence their predisposition for different sports and, potentially, success in their chosen sport. Studies have shown that combinations of multiple SNPs in dopamine-related genes can collectively enhance traits such as motivation, reward sensitivity, and stress resilience, which are crucial for athletic performance [109,110,111]. Recognizing the polygenic nature of these traits is essential to gain a more comprehensive understanding of how genetic factors contribute to athletic ability.

In addition to single gene effects, interactions between different polymorphisms (SNP–SNP interactions) play a critical role in shaping athletic performance. These interactions can modulate dopamine pathways in complex ways, influencing phenotypic outcomes beyond what individual SNP effects would predict. For example, epistatic interactions between polymorphisms in dopamine receptor genes (e.g., *DRD2* and *DRD4*) and genes involved in dopamine metabolism (e.g., *COMT* and *DAT1*) have been shown to influence behaviors related to motivation and stress response [112,113].

For example, the *DRD2* gene encodes the D2 subtype of the dopamine receptor, which plays a crucial role in the modulation of neurotransmission within the brain’s reward system. Variations in the *DRD2* gene, such as the Taq1A polymorphism, have been associated with differences in receptor availability, influencing behaviors related to reward sensitivity and motivation [114,115]. Similarly, the *COMT* gene, which is responsible for the enzymatic degradation of dopamine, particularly in the prefrontal cortex, is known for its Val158Met polymorphism, a variant studied extensively for its impact on dopamine regulation. This polymorphism affects the enzymatic activity of *COMT*, with the Met allele associated with lower enzyme activity, leading to higher dopamine levels in the prefrontal cortex and influencing cognitive functions (e.g., decision-making and executive control) [116,117,118].

Polymorphisms in the *DAT1* gene also play an important role in dopamine regulation, as *DAT1* controls dopamine reuptake from the synaptic cleft, thereby influencing its availability for synaptic transmission. Variations in this gene, such as the 40-base-pair variable number tandem repeat (VNTR) in the 3′ untranslated region (3′ UTR), particularly the 9-repeat (9R) and 10-repeat (10R) alleles, affect dopamine clearance rates and behaviors related to impulsivity and attention [119,120,121,122]. The 10R/10R genotype, often associated with altered transporter density, has been linked to increased availability and decreased synaptic dopamine [123,124]. Collectively, these genetic factors could contribute to individual differences in dopamine function, which can manifest as behavioral tendencies towards certain personality types, particularly those relevant to athletic performance.

### 3.2. Epigenetic Influence on Dopaminergic Gene Expression and Athletic Performance

Epigenetic mechanisms, such as DNA methylation, histone modification, and the activity of non-coding RNAs, play a crucial role in regulating gene expression. These changes are heritable and occur without altering the underlying DNA sequence [125,126]. Environmental factors such as stress, nutrition, and physical activity can regulate these mechanisms, leading to cellular function and behavior changes. Specifically, DNA methylation involves the addition of a methyl group to the fifth carbon atom of the cytosine nucleotide in DNA [127], typically at CpG sites, resulting in the silencing of gene expression [128,129]. This pathway can have a direct impact on cellular function and behavior. Dysfunction in DNA methylation has been associated with a variety of human diseases [130], such as type 1 diabetes [131], drug-induced diseases [132], multiple sclerosis [133], Crohn’s disease [134], or psychiatric disorders [135,136,137].

In neuroscience, epigenetic mechanisms are essential for modulating gene expression in the nervous system, influencing downstream processes like neuronal plasticity, development, and behavior [127,138,139]. These changes impact key brain functions, such as learning and memory, and can be shaped by external factors affecting dopamine pathways and related behaviors critical to athletic performance [25,140,141,142]. In the dopaminergic system, epigenetic modifications can regulate gene expression in neurotransmission, affecting dopamine levels and receptor activity [143,144].

For example, methylation of the *DAT1* gene promoter has been associated with altered dopamine transporter expression, which can significantly affect behaviors that underpin athletic performance, such as impulse control, motivation, and reward sensitivity [139,145]. Studies have shown that hypermethylation of the *DAT1* promoter region typically results in decreased dopamine transporter expression, leading to increased dopamine levels in the synaptic cleft [107]. Increased *DAT1* availability, in turn, is associated with lower extracellular dopamine levels and a reduced signal-to-noise ratio, allowing for faster neural response thresholds [146,147]. This epigenetic regulation is particularly relevant in sport, where optimal dopamine availability is essential for maintaining high levels of motivation, effectively processing rewards, and achieving peak behavioral and performance outcomes [148,149,150,151]. However, more research is needed to understand these mechanisms in the athlete model. Similarly, hypermethylation of the *DRD2* gene has been associated with reduced receptor availability, which could affect reward processing and motivational behaviors—traits critical to athletic success [152].

Histone modifications, including acetylation and methylation, also play a key role in regulating gene expression by altering the chromatin structure, making DNA more or less accessible for transcription. These modifications can affect the expression of genes involved in dopamine synthesis, transport, and receptor signaling, thereby influencing dopaminergic activity in the brain [127]. Studies in psychogenetics have highlighted how such epigenetic processes can influence traits relevant to athletic performance, including motivation, stress resilience, and goal-directed behavior. For example, Ehlert et al. discussed how stress-induced epigenetic changes can affect cognitive and emotional regulation in athletes [25], potentially enhancing or impairing performance based on gene expression patterns. Additionally, a review by Karpova et al. highlighted that histone modifications impact synaptic plasticity [153], a key mechanism in learning and adaptation critical for long-term athletic training and success. Studies also suggest that histone acetylation enhances cognitive flexibility, a critical trait for decision-making under pressure, which may benefit athletes [154,155].

### 3.3. Gene–Environment Interactions in Athletic Performance

The interaction between genetic predispositions and environmental factors is fundamental to understanding the regulation of dopamine pathways. However, there has been limited research on how these interactions specifically affect athletes. As underlined previously, environmental influences such as stress, diet, and physical activity can induce epigenetic changes, impacting gene expression and potentially providing health benefits or influencing behavior [156,157,158]. For example, chronic stress can modify DNA methylation patterns in the brain, particularly in genes involved in the stress response and dopamine regulation, potentially disrupting dopaminergic function [143].

Gene–environment interactions significantly affect personality traits and athletic performance. While genetic polymorphisms may predispose athletes to specific behaviors, environmental influences like training, coaching, and psychological interventions can modify these tendencies. For example, an athlete with a *COMT* Met/Met genotype could benefit from a stress management strategy to improve performance in high-stress situations.

Physical activity, a critical environmental factor, has been shown to induce beneficial epigenetic modifications [157]. Regular exercise can reduce DNA methylation in genes associated with dopamine production and enhance the expression of brain-derived neurotrophic factor (*BDNF*), which supports neuroplasticity and cognitive function [159,160,161]. These epigenetic changes can enhance dopamine signaling, which is crucial for maintaining motivation, resilience, and overall well-being in athletes. In addition, exercise has been shown to induce significant gene expression changes in skeletal muscle, triggering structural and metabolic adaptations for athletic development [162]. For example, acute exercise has been shown to induce hypomethylation of critical genes, such as *PPARGC1A*, *PDK4*, and *PPARD* in skeletal muscle, which is associated with increased gene expression and improved muscle function [162]. These findings suggest that DNA hypomethylation is an early event in contraction-induced gene activation and underscores the importance of exercise-induced epigenetic changes in optimizing physical performance.

Recent studies further demonstrate that a single aerobic exercise session, when combined with dietary supplementation, can significantly alter leukocyte DNA methylation and mRNA expression [163]. Specifically, one hour of cycling reduced both global and *PPARGC1A* DNA methylation and decreased *DNMT3A*, *DNMT3B*, and *PPARGC1A *mRNA expression. Interestingly, the duration of supplementation also played a role. While a 4-week supplementation period did not significantly affect global DNA methylation, more prolonged supplementation, such as 6 months with omega-3 polyunsaturated fatty acids (n-3 PUFA), led to notable changes, including decreased *LINE*-*1* DNA methylation, which may help normalize methylation levels in specific populations. In addition, the type of fatty acid supplementation produced different effects on DNA methylation; n-3 PUFA supplementation increased methylation at a CpG site in the *IL6* promoter, whereas extra virgin olive oil (EVOO) supplementation decreased it. Similar trends were observed for *DNMT1* mRNA expression and at several CpG sites in the *TNF* promoter. These epigenetic changes correlated with physiological markers related to exercise performance, inflammation, and oxidative stress, suggesting functional consequences. However, little has been discussed about how these epigenetic changes translate into tangible improvements in athletic performance. More longitudinal studies are needed to understand the long-term effects of exercise and supplementation on epigenetic modifications in athletes.

Moreover, different training modalities, such as high-intensity interval training (HIIT) and endurance exercise, may differentially influence epigenetic modifications in dopamine-related pathways. HIIT, characterized by short bouts of intense exercise followed by periods of rest, has been shown to increase dopamine availability and enhance dopamine signaling in the brain. For example, Tyler et al. demonstrated that six weeks of HIIT resulted in increased dopamine D2 receptor density and enhanced dopamine signaling in the brains of animal models [164]. This upregulation may lead to improved motivation, reward sensitivity, and cognitive function, which may be beneficial for athletes involved in sports that require rapid decision-making and explosive power.

In contrast, endurance training, involving prolonged moderate-intensity exercise, has been associated with various epigenetic changes. For instance, Voisin et al. reported that endurance exercise can lead to DNA methylation changes in genes involved in energy metabolism and mitochondrial function, which may indirectly affect dopamine synthesis and availability [165]. Seifert et al. [166] reported that endurance training increased BDNF levels in humans, which may influence dopamine neuron survival and function and contribute to improved mood and cognitive function. While direct evidence linking endurance training to epigenetic changes in dopamine-related genes is limited, these metabolic adaptations may influence dopamine production and signaling.

These results suggest that the type and intensity of physical activity may impact dopamine-related pathways, thereby influencing psychological traits and athletic performance. Tailoring training programs to promote desirable adaptations offers a tangible pathway to optimize both physical and mental aspects of performance. Personalized training programs that consider an athlete’s genetic and epigenetic profile could enhance motivation, focus, and resilience, contributing to overall athletic success.

These findings highlight the role of lifestyle interventions, such as regular exercise and supplementation, in modulating DNA methylation patterns and gene expression. The duration of these interventions is critical, as longer-term supplementation appears to have more pronounced effects. The long-term regulation of gene expression by these epigenetic mechanisms provides a molecular memory of gene–environment interactions. This highlights the potential for personalized training and nutrition programs considering an athlete’s genetic and epigenetic profile.

Incorporating lifestyle factors into strategies to optimize physical and mental health outcomes is critical [167]. Understanding dopamine’s genetic and epigenetic influences provides a framework for exploring how these biological factors translate into behavioral and psychological traits. Dopamine’s role in modulating reward sensitivity, motivation, and cognitive function underscores its importance in shaping the psychological traits contributing to athletic performance. However, much remains to be learned about how these mechanisms operate in the context of elite sport.

## 4. Dopamine and Psychological Factors in Athletic Performance

### 4.1. Psychological Traits and Their Impact on Sports Success

Psychological traits significantly influence sports performance, with dopamine playing a central role in the cognitive-motivational reward mechanisms that encourage engagement in physical activity (PA) [14,168]. Dopamine can influence motivation to engage in PA [169,170], and its role in motor function is well established [171,172]. Successful cognitive control, supported by dopamine, is another component of persistent engagement in PA [173]. Further evidence of the role of the striatal dopamine system comes from associations between dopamine depletion and motor deficits observed in Parkinson’s disease [174,175].

However, little discussion has been given to how dopamine-related psychological traits specifically contribute to athletic success in different sporting disciplines. A possible explanation for this might be the complex interplay between genetic, environmental, and psychological factors, which makes it challenging to isolate the effects of dopamine alone.

While physiological and anthropometric aspects have traditionally dominated talent identification in sports, there is increasing recognition of the importance of psychological factors in talent development [5,176,177]. Understanding these psychological traits and their neurobiological underpinnings could provide valuable mechanistic insights, as the basis for developing more effective talent identification and training programs.

### 4.2. Dopamine-Related Personality Traits and Their Impact on Athletic Success

Dopamine activity has been linked to differences in personality traits in several studies [178,179,180,181,182]. These traits, such as impulsivity, sensation-seeking, and motivation, are particularly relevant in the context of sport. For example, impulsivity, often characterized by a tendency to act without adequate forethought, has been closely linked to dopaminergic activity, mainly through the D2 dopamine receptor. Polymorphisms in the *DRD2* gene, such as the Taq1A variant, have been associated with higher levels of impulsivity and risk-taking behavior [183], traits that are critical in sports that require quick decision-making and risk assessment. However, associations between dopamine-regulating genes and broad personality phenotypes reveal inconsistent results. These inconsistencies may reflect environmental factors (e.g., parental support, life events) that drive epigenetic changes in personality, despite similar genotypes. This phenomenon, known as “phenotypic plasticity” [184], suggests that interactions with the environment can shape genotype expression, resulting in diverse personality patterns [185,186]. Despite these findings, there has been limited research on how specific dopamine-related genetic variations interact with environmental factors in athletes. A possible explanation for this gap is the complexity of conducting longitudinal studies that account for genetic and environmental variables in athletic populations.

The interaction between genetically conditioned temperament and environmentally conditioned character influences an athlete’s motivation to participate in, and persist with, sports training, as another pathway towards achieving athletic success. For example, the novelty-seeking or “risk-taking” trait has been linked to genetic variations in dopamine receptors, including the D2 and D4 subtypes, which modulate dopamine neurotransmission and influence behavior and executive function [187,188]. Understanding these interactions may help design personalized training programs that consider genetic predispositions and unique environmental factors, potentially enhancing motivation and performance for a given athlete.

### 4.3. Dopamine and Cognitive Function in Athletic Performance

Exercise has been shown to increase dopamine levels in humans [189] and rat striata [190,191]. The relevance of dopamine in sports is significant, as it potentially influences an athlete’s drive, focus, and resilience—traits essential for achieving peak performance [14,192]. Dopamine pathways facilitate the motivation to engage in and sustain intense physical activity, contributing to the mental and physical fortitude necessary for athletic success [14,193]. Additionally, dopamine’s role in motor control underscores its importance in executing the precise movements required in various sports [193,194,195,196].

Research has emphasized the importance of dopamine in enhancing the cognitive benefits associated with exercise [189]. In a study of 297 adolescents (13–17 years old) genotyped for SNPs and VNTRs related to the dopamine system, participants completed a graded exercise test to exhaustion and a control session of seated cartoon watching on separate days [197]. As assessed by a modified Rogers and Monsell task, cognitive flexibility improved significantly with exercise, as indicated by reduced switching costs. This enhancement was linked to the A allele of the *DAT1/SLC6A3* polymorphism (rs46000), which affects dopamine reuptake. Individuals with the A allele benefited more from exercise than those with the C/C genotype, possibly due to differences in resting-state connectivity, with A allele carriers having better cognitive stability and less optimal cognitive flexibility, as proposed by Cools and D’Esposito [198]. This notion is further supported by Cummins et al. [199], who found reduced cognitive stability in C allele carriers.

Berse et al.’s findings [197] suggest that physical exercise may improve cognitive flexibility in A allele carriers by enhancing their resting-state connectivity. However, little discussion has been given to how individual genetic variations in dopamine-related genes may modulate the cognitive benefits of exercise in athletes. Further research is needed to explore how these genetic factors interact with exercise to improve cognitive function relevant to athletic performance.

In addition, another study showed that regular physical activity improves cognitive function and reduces the risk of cognitive decline [189]. In this study, 27 healthy volunteers showed a 20% faster rate of vocabulary learning after intense anaerobic exercise, as compared to low-impact aerobic running or rest. This improvement was associated with increased brain-derived neurotrophic factor (BDNF) and catecholamine levels, particularly dopamine and epinephrine. Sustained levels of BDNF correlated with better short-term learning success. In contrast, higher levels of dopamine and epinephrine were associated with improved medium- and long-term retention of new vocabulary, highlighting the critical role these molecules play in mediating the cognitive benefits of exercise. These findings underscore the potential of exercise to enhance cognitive functions important for athletic performance, possibly mediated by dopaminergic activity.

### 4.4. Practical Applications of Dopamine Research in Enhancing Athletic Performance

Understanding the role of dopamine in psychological traits and cognitive function has practical implications for improving athletic performance. Coaches and sports psychologists could develop personalized training and intervention programs by recognizing the genetic and neurobiological factors influencing motivation, concentration, and cognitive flexibility. For example, interventions that increase dopaminergic activity, such as high-intensity interval training (HIIT), or specific nutritional strategies, such as tyrosine supplementation, could be used to improve motivation and cognitive function in athletes [200,201].

In addition, psychological interventions designed to improve cognitive control and/or resilience could leverage the effects of the dopaminergic system in these processes. Techniques such as mindfulness training, cognitive-behavioral strategies, and stress management may also help to optimize the dopamine response, thereby reinforcing a psychological response to achieve athletic goals and success [202,203]. Furthermore, understanding the interaction between genetic predispositions and environmental factors underscores the importance of a supportive training environment. Coaches and support staff could create conditions that promote positive psychological traits, such as motivation and resilience, thereby enhancing the athlete’s natural dopaminergic function.

While sports psychogenetics holds great promise, it is still in its early stages and not yet widely applied in practice. Knowledge of genotypes associated with desirable personality traits and psychological abilities could provide another tool for talent identification, athlete selections, and the creation of personalized training programs. Athletes with specific genetic profiles might more effectively cope with stress, recover faster, and manage emotions better—factors critical to achieving high-performance levels [204,205]. This genetic information could help sports psychologists develop more tailored mental support strategies for athletes.

However, it is essential to recognize that the practical applications of genetic information in sports are currently limited. Ethical considerations such as privacy, consent, and potential discrimination must be carefully managed [206,207]. Therefore, while the potential benefits are significant, any practical applications must be approached cautiously and within an appropriate ethical framework. Further research is needed to understand the implications and fully translate these findings into practice.

## 5. Dopamine’s Role and Athletic Performance

### 5.1. Dopamine’s Impact on Athletic Performance and Motivation

Dopamine is critical in regulating physical endurance and motivation in athletes, making it a central component of athletic performance. Physical activity affects central dopaminergic, noradrenergic, and serotonergic systems and increases neurotransmitter activity [200,208]. This interaction suggests a bidirectional relationship between PA and dopamine synthesis [169,170,192]. What is lacking, however, is a detailed discussion of how these bidirectional effects specifically influence different types of athletic activities across various sports disciplines. A possible explanation lies in the diversity of sports and the unique physiological and psychological demands each imposes, making it challenging to generalize findings across sports.

Existing literature emphasizes that PA and exercise benefit dopamine synthesis, with most studies reporting increased dopamine levels or receptor availability following exercise [209,210,211]. This supports the role of PA in improving mental health by improving cognition [212,213,214], increasing brain-derived neurotrophic factor (BDNF) levels [215], and promoting brain plasticity [213]. Additionally, habitual PA is positively associated with dopamine-related gene expression [216], suggesting that consistent exercise may promote elevated dopamine levels, thereby contributing to mental well-being and potentially reducing symptoms of depression, anxiety, and other mental health problems [208,217,218,219,220].

Recent studies shed light on the complex relationship between dopamine pathways and athletic performance. Dopamine pathways, particularly the mesolimbic system, reinforce goal-directed behaviors, which are crucial for training adherence and long-term sporting success [221]. Despite these insights, there has been limited exploration of how specific dopaminergic pathways interact with different training regimens and competitive environments to influence performance outcomes. A possible explanation might be the complex nature of neurobiological interactions that vary significantly among individuals and sports.

A systematic review published in 2021 emphasized the bidirectional effects of physical activity on dopamine levels, highlighting how regular exercise can improve dopaminergic function across age groups. The review also suggested that increased dopamine activity could improve motivation and resilience in athletes, particularly those involved in endurance sports [192]. In addition, a 2010 study explored the role of dopamine in the recovery process and found that variations in dopaminergic activity may influence the rate at which athletes recover from intense physical exertion, thereby affecting overall performance outcomes [222].

Moreover, exercise induces significant adaptations in dopaminergic circuits, resulting in a hyperdopaminergic state that enhances dopamine release in the striatum during exposure to non-exercise stimuli, including aversive stressors [223]. Sensitized dopamine release promotes the activation of neurons expressing D1 receptors, which are associated with reward and stress resilience, over neurons expressing D2 receptors, which are associated with aversion and stress vulnerability [223]. These neural adaptations suggest that regular physical activity not only enhances dopaminergic function but also modulates the brain’s response to stress, thereby promoting resilience and sustained high performance in athletes. However, more focused research is needed to understand how these adaptations vary across different sports and individual athlete profiles.

### 5.2. Dopamine Pathways and Their Role in Athletic Performance

Dopamine operates through four major pathways present in the human brain: the mesolimbic, mesocortical, nigrostriatal, and tuberoinfundibular pathways [224]. These pathways uniquely contribute to motor control, cognitive function, and reward processing. The mesolimbic pathway, often referred to as the brain’s reward circuit, is activated during pleasurable activities, including physical exercise [225]. This pathway extends from the ventral tegmental area (VTA) to the nucleus accumbens (NAc) and plays a central role in reinforcing behaviors that are perceived as rewarding [221]. For athletes, the dopamine surge experienced during exercise can enhance performance and reinforce this behavior, making regular training a rewarding experience.

Dopamine also plays a pivotal role in the mesocortical pathway, influencing cognitive functions such as decision-making and memory by connecting the VTA to the prefrontal cortex [226]. Stimulants that induce motivation and reward (sex, food, water, drugs, listening to music) increase dopamine release [227,228]. The nigrostriatal pathway, which connects the substantia nigra to the striatum, is important for controlling motor function, learning motor skills, and planning [228], while the tuberoinfundibular pathway, linking the hypothalamus to the pituitary gland, regulates hormone release, affecting processes such as libido and lactation [229]. However, there has been little discussion about how these distinct dopamine pathways interact with the specific demands of different sports disciplines.

It is important to note that dopamine is not only a key player in the reward system, but it also interacts with other monoamines (e.g., norepinephrine, serotonin) to regulate brain functions that are enhanced by exercise [217,230]. This interaction underlies the benefits of PA for mental health, where dopamine release during acute exercise can improve mood and cognitive function. However, there are conflicting reports regarding the extent and conditions under which dopamine levels increase with exercise, suggesting that the relationship is not entirely straightforward. For example, Tedjasaputra et al. found that while dopamine plays a role in improving pulmonary gas exchange, its inhibition may decrease exercise performance [231]. Interestingly, Cordery et al. observed that acute dopamine/noradrenaline reuptake inhibition enhances human exercise performance only under warm conditions, but not in temperate environments, highlighting how environmental factors can modulate dopaminergic effects on performance [232,233]. Roelands et al. further demonstrated that the efficacy of dopaminergic agents is dose-dependent, adding another layer of complexity to the relationship between dopamine and exercise performance [234]. These examples illustrate that the relationship between dopamine and exercise performance is influenced by various moderating factors such as environmental conditions, individual genetic makeup, and the specific type of physical activity performed.

While the specific characteristics of exercise—such as frequency, intensity, and context—may influence these outcomes [214], the overall behavior of engaging in PA is paramount to achieving mental health benefits [14,209,211,235]. This has been observed in previous studies, which emphasize that dopamine levels only increase when a threshold physical intensity is reached [190,236]. In addition, emerging evidence suggests that acute bouts of aerobic exercise can effectively increase dopamine, serotonin, and norepinephrine concentrations in both animal models and humans, including individuals with Parkinson’s disease [237,238,239,240]. However, it remains uncertain whether these peripheral neurotransmitter increases successfully cross the blood–brain barrier to influence central neurotransmitter levels [241]. Given the role of low dopamine synthesis in various mental health problems [242], these findings suggest that prescribing exercise may be an effective strategy for treating such conditions.

Dopamine’s influence on athletic performance and motivation is profound. As the “pleasure neurotransmitter”, dopamine increases motivation, executive function, enthusiasm, focus, and reward-seeking. It boosts determination and mental resilience, helping athletes push their limits and achieve peak performance. High dopamine levels are associated with increased drive and goal-oriented behavior—traits essential for athletes. For example, elite athletes often show increased dopaminergic activity, which supports sustained effort and resilience in training and competition [4].

Dopamine’s role in the brain’s reward system is a key component of the motivational circuitry and, in the context of sport, seems necessary to engage in, and persist with, rigorous training regimens. The release of dopamine in response to rewarding stimuli, such as achieving a personal best or winning a competition, reinforces the behaviors that lead to such outcomes and ensures that athletes remain committed to achieving their goals over time [221,243]. Anticipation of reward activates dopaminergic pathways, particularly in the nucleus accumbens and prefrontal cortex, which are associated with increased effort and goal-directed behavior. This anticipation is crucial for athletes, as their motivation to train and improve often depends on the expectation of future rewards, whether intrinsic, such as personal satisfaction, or extrinsic, such as trophies and recognition [244,245].

In addition to its effects on motivation, dopamine also contributes to stress resilience in athletes [246,247,248]. High dopamine levels are associated with an increased ability to cope with competitive sports’ physical and mental stressors. The dopaminergic system helps regulate stress responses, allowing athletes to maintain focus and composure under pressure, which is critical for success in high-stakes competitions [223]. However, there has been little discussion of how individual differences in dopaminergic regulation affect the ability of athletes to cope with stress and recover from competitive pressure. A possible explanation may be the multifaceted nature of stress and its interaction with multiple neurotransmitter systems beyond dopamine alone. Moreover, dopamine’s influence extends to how athletes cope with setbacks or failure [14]. A well-functioning dopaminergic system allows for adjusting motivation and reward expectations which, in turn, might help athletes persist in their efforts despite obstacles. This resilience is critical to overcoming challenges and achieving long-term success in sport [249].

While dopamine is undeniably integral to athletic performance and motivation, the nuanced ways it interacts with various physiological and psychological factors warrant further exploration. Understanding these complex interactions may provide deeper insights into optimizing athletic training and performance strategies.

### 5.3. Genetic and Epigenetic Influences on Elite Athletic Performance

Understanding the relationship between genetics and athletic performance has become a significant focal point in sports science. Identifying the *ACE* I/D polymorphism in 1998 was a breakthrough in sports genetics, linking it for the first time to human physical performance [250]. Since then, the number of identified genetic markers associated with athletic performance has grown significantly. For example, a study from 2009 [251] identified 232 genes associated with athletic performance, which increased to 251 genes by March 2023 [252]. However, there has been little discussion of how these numerous genetic markers interact with each other, and with environmental factors to comprehensively influence athletic performance, possibly due to the complexity and polygenic nature of athletic traits, which makes it difficult to isolate the effects of individual genes.

The importance of considering polygenic profiles in elite athletes cannot be overstated. Athletic performance is a complex trait influenced by a constellation of genetic polymorphisms, each contributing incrementally to the overall phenotype. Recent genome-wide association studies (GWASs) have identified associations between multiple SNPs and athletic traits [253]. For example, the combined effect of SNPs in dopamine-related genes may influence not only physical attributes, but also psychological traits essential for performance under pressure.

Moreover, SNP–SNP interactions add another layer to the genetic basis of athletic performance. These interactions can result in non-additive effects, where the combined influence of two or more SNPs results in a phenotype that is different from the sum of their individual effects. This phenomenon, known as epistasis, has been observed in studies examining the interaction between dopamine receptor genes and genes involved in dopamine synthesis and metabolism [112]. Understanding these interactions is critical to elucidating the genetic mechanisms underlying athletic success.

These genes influence endurance, muscle strength, and power, underscoring the genetic complexity underlying physical fitness. Like many human traits, athletic performance traits are highly polygenic, meaning numerous polymorphisms influence them, each contributing a small effect. In addition, these traits often exhibit pleiotropy, in which individual polymorphisms can influence multiple traits [109,254]. This polygenic and pleiotropic nature complicates the identification of specific genetic factors that can be targeted to improve performance, highlighting the need for more integrative and multifaceted research approaches. This complexity is mirrored in psychological traits, where many polymorphisms, particularly in the serotonergic and dopaminergic systems, influence behavior and personality [111,112,255,256]. These markers underscore the complexity of genetic and environmental interactions and the polygenic nature of many traits important to sport [5]. So far, however, there has been little discussion about how these genetic and psychological factors collectively contribute to an athlete’s success, suggesting a gap in holistic models of athletic performance.

In addition to genetic markers that influence physical traits, it is essential to understand how genetics influence psychological aspects of athletic performance. To date, there has been little discussion of the specific polymorphisms that influence mental resilience and motivation in athletes. Psychogenetics, an emerging interdisciplinary field, explores how genetic factors influence mental functioning and behavior, particularly in sports. This field integrates molecular biology, psychology, and exercise science to understand how these genetic factors contribute to athletic performance, motivation, and mental resilience. A possible explanation for the limited discussion in this area might be the nascent stage of psychogenetics and the challenges associated with studying complex psychological traits in diverse athletic populations. Given the polygenic and pleiotropic nature of psychological phenotypes, a wide range of polymorphisms beyond those affecting physical performance must be considered to fully understand how genetics shape psychological traits relevant to athletes [113,257,258]. This multifaceted approach is necessary to capture the intricate interplay between multiple genes and their combined effect on an athlete’s psychological profile.

As a complementary layer to genetic influence, epigenetics bridges genetic and environmental interplay, contributing to an athlete’s predisposition for success [25,259]. Epigenetic modifications, such as DNA methylation, are important in linking genetic factors with environmental exposures, such as training, to influence gene expression. This interplay can influence synaptic plasticity and learning processes, critical for brain adaptation and skill acquisition in response to training [154,260,261]. However, there has been little discussion of how specific epigenetic modifications interact with specific training regimens to optimize performance outcomes. The variability in training protocols and the individualized nature of epigenetic responses among athletes offer one plausible explanation.

Exercise has been shown to induce significant changes in DNA methylation profiles, which regulate gene expression and facilitate adaptation to environmental conditions. Still, findings on how exercise affects global DNA methylation levels, particularly in the blood, are inconsistent [262]. Some studies have reported DNA hypomethylation [263,264] or hypermethylation [265,266] in peripheral blood mononuclear cells due to physical activity. In contrast, others have found no significant effect of exercise on global DNA methylation, regardless of variables such as age, type of exercise, fitness level, or cell type [267,268]. A complementary study on recreationally active young men, who underwent eight weeks of resistance training [268], found that while there was no overall change in global methylation levels, the methylation profile itself was significantly altered, with thousands of specific sites either methylated or demethylated. This suggests that while global methylation remains stable, exercise can induce substantial changes in specific genomic regions. This inconsistency suggests that the relationship between exercise and DNA methylation is influenced by multiple factors (such as tissue types analyzed, DNA methylation analysis methods, sample size, or gender differences), and more standardized methodologies are needed to clarify these effects [165,269,270].

Research distinguishes between acute and chronic DNA methylation responses to exercise. A study by Barrés et al. reported that acute exercise decreased whole genome methylation in the skeletal muscle of sedentary men and women. This decrease was prominent in selected genes such as *PPARGC1A*, which encodes peroxisome proliferator-activated receptor gamma coactivator 1-alpha (PGC-1α), a key transcriptional coactivator involved in the regulation of genes involved in energy metabolism, as well as *PDK4* and *PPARD* [162]. This hypomethylation was associated with increased gene expression, suggesting that DNA hypomethylation is an early event in exercise-induced muscle gene activation. Bajpeyi et al. also observed inter-individual variability in the methylation response following exercise, which they attributed not only to genetic differences but also to individual epigenetic responses, underscoring the complexity of exercise-induced epigenetic modifications [271]. They found that endurance exercise reduced nucleosome occupancy around the −260 nucleotide region in the *PPARGC1A* promoter, a key regulatory site for DNA methylation. This reduction correlated with increased *PGC1α* mRNA expression in the skeletal muscle of healthy men after a single session of endurance exercise. These findings highlight the need for personalized training programs that consider genetic and epigenetic profiles to optimize performance and adaptation.

Some studies have not found significant changes in global DNA methylation levels after exercise, but have reported variations in methylation patterns of CpG islands in the promoter regions of specific genes. For example, Robson-Ansley et al. observed no changes in global DNA methylation after a prolonged aerobic exercise session that included 120 min of running at 60% VO_2max_ followed by a 5 km time trial [272]. This result may indicate that changes in DNA methylation were minimal or that changes in hyper- and hypomethylation were relatively balanced. However, the limited number of participants in this study may have affected the ability to detect more subtle methylation changes. Therefore, a larger sample size might be necessary to capture the full extent of exercise-induced epigenetic modifications.

Chronic responses to exercise present a different picture, as shown in studies examining long-term training effects. A recent study [273] revealed that after a 7-week resistance training program, there were significant changes in DNA methylation at over 18,000 CpG sites, with a mix of hypo- and hypermethylation. These epigenetic modifications persisted even after detraining, suggesting that muscle cells retain an “epigenetic memory” of previous training stimuli. Lindholm et al. conducted an interesting study investigating the epigenetic effects of endurance training on skeletal muscle [274]. In this work, sedentary volunteers trained one leg exclusively for three months, performing four 45-min weekly sessions. This setup allowed the researchers to isolate the effects of exercise on DNA methylation by comparing the trained leg to the untrained leg within the same individual. The results revealed nearly 5000 sites of altered DNA methylation and changes in the expression of approximately 4000 genes in the trained leg. Notably, no significant changes in global methylation levels were identified, indicating that endurance training induces specific epigenetic modifications that correlate with gene expression changes related to muscle adaptation and growth [274]. Training and sex were identified as significant determinants of variability in methylation on autosomal DNA. These findings underline both training intensity and biological sex as factors shaping epigenetic responses.

An intriguing area is sex differences in epigenetic responses to exercise, given the role of DNA methylating (DNMT) and demethylating (TET) enzymes. Early studies suggest that the expression of these enzymes may differ between the genders, affecting muscle adaptation to exercise. A meta-analysis of 16 studies [275] identified 478 loci that undergo methylation changes following either acute (single bout) or chronic exercise, such as walking, cycling, and Tai Chi. The study found that DNA methylation generally decreased at 60% of these loci following exercise, with more significant changes observed in older individuals. These findings suggest that exercise can significantly impact epigenetic markers, potentially reversing age-related changes in DNA methylation. Furthermore, the meta-analysis highlighted that age, sex, and tissue type can influence the magnitude of methylation changes, indicating the nuanced nature of the epigenetic response to exercise. This underscores the importance of considering demographic and biological variables when studying epigenetic adaptations to exercise, paving the way for more tailored and effective training programs.

Despite growing research on epigenetics in athletes, studies on how DNA methylation relates explicitly to personality traits in this group are still limited. Epigenetic modifications, such as DNA methylation, can influence gene expression based on genetic polymorphisms and environmental factors, including exercise. Nevertheless, there has been little discussion of the specific pathways through which epigenetic changes influence personality traits critical for athletic performance, such as resilience, motivation, and stress management. While many studies have documented exercise-induced epigenetic changes, few have focused on their potential impact on personality traits among athletes. For example, Grzywacz et al. found significant associations between DNA methylation in the *DAT1* gene and personality traits like lower openness and higher neuroticism in martial artists [276]. Our own research [26] observed higher methylation levels in the *DAT1* gene’s promoter region in martial artists compared to non-athletes, correlating with traits such as extraversion. We also found a more significant number of methylated CpG islands in MMA than in non-athletes. Expanding on this work, it would be fruitful to study how DNA methylation might influence personality traits that are important for psychological resilience and athletic performance.

Overall, these studies underscore the complex and dynamic nature of epigenetic modifications in response to exercise, where a myriad of factors such as the type of exercise, individual genetic makeup, and even sex all play a regulatory role. Nevertheless, there has been little discussion about how these epigenetic changes can be harnessed to enhance specific performance-related traits in athletes. In the future, overcoming these methodological challenges will be critical to advancing our understanding of optimizing exercise-induced epigenetic changes for health and performance in athletes.

## 6. Genetic Polymorphisms Influencing Dopamine and Athletic Performance

Despite significant advances in understanding the genetics of physical fitness, research targeting the genetic basis of psychological traits in athletic populations remains limited, although psychological profiling has been undertaken [277,278,279]. To provide a clearer picture of the current landscape, Table 1 presents a comprehensive review of studies that have examined the relationship between personality traits and dopamine-related genetic polymorphisms in athletes. So far, however, there has been little discussion about how these genetic factors interact with psychological traits in a meaningful way to regulate athletic performance or development. It is already known that several key genes are integral to dopaminergic function and influence various aspects of athletic performance and personality traits. These genes affect dopamine availability, receptor sensitivity, and neuronal growth, which are crucial for motivation, reward processing, and stress resilience, all essential traits in sport. Nevertheless, the table highlights a notable gap in the literature.

Different sporting disciplines place varying demands on psychological traits, and dopamine-related genetic polymorphisms can influence how athletes meet these demands. For example, in combat sports such as mixed martial arts (MMA), where stress resilience and quick decision-making are critical, studies have shown that certain dopamine-related polymorphisms are associated with personality traits deemed valuable to expressing performance. In a study involving Polish MMA athletes, the deletion polymorphism of the *DRD2* gene (rs1799732) was associated with lower reward dependence and harm avoidance [282,291]. Lower reward dependence may contribute to greater independence and reduced need for external reinforcement, which may enhance concentration and self-confidence during competition. In addition, these traits likely allow athletes to remain calm under pressure and make quick decisions, which are essential for success in such high-stress combat situations.

Similarly, sustained motivation and psychological resilience are essential in endurance sports such as Ironman triathlons. Van Breda et al. [295] found that ultra-endurance athletes with the Met/Met genotype of the *COMT* Val158Met polymorphism showed higher novelty-seeking and lower harm avoidance compared to controls. Higher novelty-seeking may influence endurance performance by increasing motivation for prolonged effort and adaptability to changing conditions. In contrast, our study on Polish male combat sports athletes revealed that Val/Val (G/G) carriers had lower novelty-seeking and higher self-management scores, indicating better focus and stress resilience under pressure [283]. This genotype may confer advantages in sports that require sustained concentration and emotional control.

Team sports such as football (soccer) also show associations between dopamine-related genes and personality traits. McAuley et al. reported that English male football players with the A/A genotype of the *DRD3* rs167771 polymorphism showed higher levels of agreeableness than those with the G allele [286]. Higher agreeableness may enhance teamwork and cooperation on the field, positively impacting team performance.

In sports that require concentration and emotional control, such as martial arts, certain *BDNF* gene polymorphisms have been associated with personality traits that enhance performance. In our previous study of Polish martial artists [64], the Val/Val (G/G) genotype of the *BDNF* gene (rs6265) was associated with higher conscientiousness and lower neuroticism compared to controls. These traits contribute to better focus and emotional stability, which are essential for success in disciplines that require discipline and precision. These examples illustrate how dopamine-related genetic variations may differentially influence personality traits that are, at least intuitively, relevant to specific disciplines in sport. Expanding this concept, it is attractive to hypothesize that athletic performance might be optimized by matching genetic predispositions with the psychological and physical demands of that sport.

While individual studies have identified associations between specific SNPs and personality traits, an integrative approach is lacking that synthesizes these findings to elucidate overarching patterns or mechanisms. Variability in study designs, sample sizes, and specific traits examined could explain this gap, which makes it challenging to aggregate consistent and coherent results. However, the complex mechanisms by which these polymorphisms translate into performance-enhancing traits remain to be elucidated. Research has highlighted the impact of genetic polymorphisms on motor skill learning, which is crucial for athletic performance.

### 6.1. Dopamine Receptor Genes and Personality Traits in Athletes

Dopamine receptor genes, particularly *DRD2* and *DRD4*, are essential in regulating dopamine’s interaction with neurons, influencing traits such as risk-taking, novelty-seeking, stress tolerance, and perseverance. These personality traits are critical to decision-making and performance in high-pressure sports environments. For example, the dopamine D4 receptor (*DRD4*) gene is interesting due to its highly polymorphic nature, especially the variable number tandem repeat (VNTR) polymorphism in exon III. This VNTR polymorphism, particularly the presence of the seven-repeat (“long”) allele, has been associated with reduced receptor binding [296], reduced gene expression, and lower cyclic adenosine monophosphate (cAMP) formation when dopamine binds to the receptor [297], compared to shorter alleles, potentially influencing novelty-seeking behavior. Despite these associations, limited research has examined how these genetic variations affect athletic behavior and performance outcomes.

In team sports such as football, where coordination and social interaction are essential, the *DRD3* rs167771 polymorphism also plays an important role. McAuley et al. [286] found that football players with the A/A genotype showed higher agreeableness, which may improve team cooperation and performance. In team sports such as rugby, Abrahams et al. found that players with the *DRD4* rs1800955 TT and CT genotypes were associated with low reward dependence, suggesting that these players may be more self-confident and less influenced by external feedback [280]. Such traits may influence how players respond to coaching and team dynamics. In addition, high harm avoidance appeared to decrease concussion risk among juniors (*p* = 0.009), but at the same time increased the risk of concussion among seniors (*p* = 0.001), suggesting that personality traits influenced by polymorphisms may modulate injury susceptibility in competitive settings.

Early studies found that a long seven-repeat allele of the D4 dopamine receptor gene (*DRD4*) was associated with higher scores on novelty-seeking, a personality trait characterized by impulsivity, exploration, and excitability [298,299]. In a study of 124 unrelated Israeli participants [298], individuals with this allele showed significantly higher novelty-seeking scores on the Tridimensional Personality Questionnaire (TPQ), which measures four domains of temperament: novelty-seeking, harm avoidance, reward dependence, and persistence. These findings, independent of ethnicity, sex, or age, were among the first to link a specific genetic variation in dopamine receptors to normal personality traits, highlighting the genetic basis of behavioral tendencies. The association between long alleles of the *DRD4* gene polymorphism and novelty-seeking was confirmed in family studies due to genetic transmission rather than population stratification [299]. Subsequent research has, however, yielded mixed results, and this implies that the relationship between *DRD4* polymorphisms and personality traits is more complex than initially thought.

In combat sports, Michalowska-Sawczyn et al. reported that Polish male combat sports athletes with the *DRD4* Ex3 l/l genotype exhibited higher conscientiousness, while those with the s/s genotype showed lower openness [288]. These personality traits may influence an athlete’s approach to training and adaptability during competition.

These findings underscore the genetic basis of behavioral tendencies like exploration and excitement, which are crucial in sports and demand quick adaptation to new challenges. However, research on the association between *DRD4* polymorphisms and novelty-seeking has yielded mixed results [178,300,301]. A meta-analysis of 20 studies with 3907 non-athletic participants [302] found no significant overall association between the *DRD4* VNTR polymorphism and novelty-seeking. Thirteen studies reported an association between longer *DRD4* alleles and higher novelty-seeking scores, while seven found the opposite. The studies showed true heterogeneity, suggesting that unknown moderators may exist, but their effects are likely weak. Similarly, a more recent meta-analysis [178] found that while the C-521T (rs1800955) polymorphism in *DRD4* was associated with novelty-seeking and impulsivity, the Ex3 VNTR polymorphism was not. Given these inconsistencies, more nuanced research approaches should consider potential moderating factors such as environmental influences, gene–gene interactions, and the specific contexts in which personality traits are expressed. According to the authors, the C-521T polymorphism, if indeed associated, could explain up to 3% of the phenotypic variance in these traits. This meta-analysis included 36 studies on the Ex3 VNTR and 11 studies on the C-521T *DRD4* polymorphism. However, there was significant between-study heterogeneity for the Ex3 VNTR polymorphism (*p* < 0.001). Inconsistencies in studies investigating the association between the *DRD4* exon III polymorphism and personality traits are likely due to a combination of demographic and methodological factors. Differences in participant demographics, such as age, sex, or clinical status, may influence the expression of personality traits, leading to variable results and, potentially, the underestimation of the strength of these associations. Methodological differences, including sample size, selection criteria, genotyping methods, and the use of different psychological assessments, also contribute to these inconsistencies. These factors suggest that the mixed results may be influenced by study-specific conditions, rather than the absence of a true genetic association. Future research should focus on theory-driven approaches, use standardized inclusion and exclusion criteria, and recruit larger, more diverse sample sizes to identify potential moderators with greater certainty.

In sports, particularly among elite athletes, these genetic variations have been suggested to play an essential role in shaping personality traits that contribute to athletic success. Building on previous findings in non-athletes [178], a study of skiers and snowboarders [293] found a significant association between the *DRD4* -521 CC genotype (rs1800955) and higher levels of sport-specific sensation-seeking. In another study by Thomson et al. (2013) involving a larger sample of skiers and snowboarders (M = 341, F = 258; Caucasian), the researchers tested 26 SNPs across eight dopaminergic and serotonergic genes for associations with both general and sport-specific sensation-seeking behavior [294]. Notably, they found a significant association between the *DRD3* rs167771 polymorphism and sensation-seeking, with lower scores associated with the G allele. This finding highlights the potential role of genetic predisposition in shaping risk-taking behavior in sports, which is critical for performance in high-adrenaline, competitive environments. However, rs1800955 (*DRD4*) polymorphism was not associated with general impulsive sensation-seeking, suggesting that these genetic effects may be context-dependent. Similarly, Michałowska-Sawczyn et al. examined the *DRD4* VNTR polymorphism in 302 physically active participants, including 200 elite male combat athletes and 102 healthy male controls, finding associations with personality traits relevant to athletic performance [288]. The study found that the *DRD4* Ex3 s/s (short allele) genotype was associated with lower openness, which reflects a preference for routine and practicality over novelty and imagination, and higher conscientiousness, which is associated with traits such as discipline, organization, and a strong sense of duty. These results suggest that while certain *DRD4* polymorphisms influence specific personality traits, their effects may vary depending on the athletic context and type of sport.

Research examining the association between polymorphisms in the *DRD2* gene, which encodes the D2 dopamine receptor, and personality traits in athletes has highlighted the significant role that genetic variations in dopamine receptors play in influencing behaviors associated with high-risk sports, particularly within the brain’s reward system. Variations in the *DRD2* gene, such as the Taq1A polymorphism, have been associated with differences in receptor density and reward sensitivity [24,303]. Specifically, individuals carrying the A1 allele (rs1800497) tend to have lower receptor availability, leading to heightened impulsivity and a greater propensity for risk-taking behaviors [304,305]. However, the extent to which these genetic variations influence actual athletic performance and risk-taking in sports remains underexplored and requires further investigation.

This trait can be advantageous in sports like extreme skiing, rock climbing, and other high-risk disciplines, where athletes must often make quick, high-stakes decisions. The A1 allele has also been implicated in reward deficiency syndrome, which can influence an individual’s pursuit of novel experiences or stimuli [306,307,308]. Among athletes, this drive may manifest as a preference for intense, high-risk environments, giving them an edge in those sports that require these features.

A study by Niewczas et al. examined personality traits and the *DRD2* rs1799732 deletion polymorphism in 85 Polish MMA athletes and 284 healthy controls [291]. The results showed that MMA athletes scored lower on harm avoidance and reward dependence and higher on self-management than controls. Lower harm avoidance indicates a greater willingness to take risks, which is beneficial in high-risk sports such as MMA. Lower reward dependence reflects less reliance on social approval and external rewards, indicating greater independence. Self-management includes discipline and emotional control, which are essential for maintaining focus under pressure. Despite these associations, the causal pathways linking *DRD2* polymorphisms to these specific personality traits in athletes are not yet fully understood.

This personality profile indicates a more independent, risk-tolerant, and self-regulated approach to competition. In addition, the *DRD2* rs1799732 deletion polymorphism was associated with lower reward dependence in MMA athletes, suggesting that this polymorphism contributes to personal independence and a reduced need for external reinforcement. This is consistent with the broader understanding of the role of dopamine in reward-seeking behavior and reinforces the importance of *DRD2* polymorphisms in shaping traits such as risk tolerance and psychological resilience in high-pressure sports.

A previous study of 109 non-athletic, healthy individuals (78 female and 31 male) examined several other polymorphisms of the dopamine receptor genes *DRD4* (VNTR Ex3 polymorphism) and *DRD2* (Ser311Cys) to investigate their association with personality traits such as novelty-seeking, harm avoidance, reward dependence, and persistence [309]. This study found no significant associations between these polymorphisms and personality traits such as novelty-seeking, harm avoidance, or persistence, as measured by the Temperament and Character Inventory (TCI).

In contrast, our study on the *DRD2* Taq1D rs1800498 polymorphism in 391 male subjects (159 athletes and 232 controls) showed significant associations with personality [285]. Of these participants, 159 were athletes actively involved in various sports disciplines (including karate, judo, boxing, wrestling, ju-jitsu, volleyball, and handball league players), and 232 were untrained controls. The athletes presented higher levels of extraversion and conscientiousness on the NEO-FFI scales than controls. In addition, the *DRD2* rs1800498 polymorphism was explicitly associated with higher conscientiousness in athletes, suggesting a potential genetic influence on traits critical for sporting success, such as discipline and organization. This is consistent with the findings of Simonen et al., who observed that *DRD2* TT homozygotes in the exon 6 C/T polymorphism were 27–33% less likely to participate in sports and physical activity, particularly among white women [310]. Similarly, Lee et al. found that the *DRD2* A1 allele of the Taq1A polymorphism (rs1800497) was associated with a decreased likelihood of sports participation during adolescence and young adulthood [311]. In addition, Flack et al. reported that the A1 allele correlated with a lower relative reinforcing value of exercise in adults [170], while a study conducted among randomly selected adult men in Poland [312] found no significant associations between physical activity levels and *DRD2* (rs6275) and *DRD4* (48-bp VNTR) polymorphisms. These mixed findings suggest that the influence of *DRD2* polymorphisms on athletic performance and participation may be context-dependent and influenced by other genetic and environmental factors.

A recent case–control genetic association study by Abrahams et al. examined the role of *DRD2* and *DRD4* polymorphisms in personality traits and concussion susceptibility in junior and senior rugby players [280]. The study found that the rs1800955 CC genotype and the combined *DRD2* (rs12364283-rs1076560)–*DRD4* (rs1800955) A-C-C allele combination were associated with a lower risk of concussion in junior players. In addition, the *DRD4* rs1800955 T allele was associated with socially distant behavior, and the TT and CT genotypes of rs1800955 were associated with low reward dependence. High harm avoidance, characterized by cautious and risk-averse behavior, was associated with lower concussion risk in juniors but increased risk in seniors. These findings highlight the complex role of genetic polymorphisms not only in influencing personality traits, but also in modulating injury risk in athletes. This research emphasizes how polymorphisms can influence athletic performance and personality traits, such as harm avoidance and reward dependence, providing insight into concussion risk and broader implications for athlete safety. Nevertheless, there has been little discussion about how these genetic associations translate into practical strategies for injury prevention and performance optimization in athletes. These results suggest that although there are only a few studies analyzing personality traits in athletes, some *DRD2* polymorphisms such as rs1799732 may influence traits such as reward dependence and cooperation ability, while other polymorphisms in *DRD4* and *DRD2* may not significantly influence personality traits and athletic performance. In further discussing the influence of genetic factors on athletes’ personality traits, a study focusing on ten specific SNPs in young soccer players revealed a significant association between *DRD3* (rs167771) and agreeableness. The study found that individuals with the A/A genotype demonstrated higher agreeableness levels than those with the G allele [286]. Other SNPs examined in the study included *HTR2A* (rs6311), *BDNF* (rs6265), *COMT* (rs4680), *DRD2* (rs1800497), *DRD4* (rs1800955), and others, but no significant associations were found with the other personality traits or mental toughness. The complexity of linking genetic factors to psychological traits is evident from this work, as only modest effects were observed and because individual SNPs tend to explain only a small fraction of the variance in personality traits, even when multiple polymorphisms are examined. While genetics plays a role in expressing traits such as agreeableness, the overall effect is modest. This highlights the need for polygenic approaches in future research, as focusing solely on individual SNPs may underestimate the total genetic contribution to personality and performance traits in athletes. Expanding the range of genetic markers studied will provide deeper insights into these influences on personality and performance traits in athletes.

Overall, while dopamine receptor genes such as *DRD2* and *DRD4* play an integral role in shaping personality traits relevant to athletic performance, the relationship is complex and influenced by multiple factors. However, there has been little discussion of how these genetic variations interact with environmental influences and training regimens to optimize athletic performance. Future research should employ integrative models that consider both genetic and environmental factors to fully understand the role of dopamine-related polymorphisms in athletic success.

### 6.2. Brain-Derived Neurotrophic Factor (BDNF) and Personality Traits in Athletes

Another gene that is essential for neuronal growth and synaptic plasticity, processes closely linked to cognitive function and emotional stability, is the *BDNF* gene. Despite its significant role, limited research has sought to describe how *BDNF* polymorphisms specifically affect personality traits relevant to athletic performance. Brain-derived neurotrophic factor (BDNF) is a neurotrophin involved in neuroprotection and neurogenesis, both critical for brain health. In humans, serum BDNF (sBDNF) levels are an indicator of brain function, particularly in the hippocampus, and higher sBDNF is generally associated with better performance on cognitive tasks [313,314,315]. Physical exercise effectively stimulates an increase in sBDNF, improving executive and memory function and reducing hippocampal atrophy, especially in older adults [316,317]. However, the response of sBDNF in athletes, especially in older populations, remains unclear and may vary due to additional stressors or different mechanisms at play [318]. A study on fourteen youth badminton athletes showed a decrease in sBDNF levels and sleep time by the end of the season. Despite this, cognitive function improved during the season, while VO_2max_ was positively correlated with sBDNF only in the preseason period. These findings suggest that multiple factors, including training intensity, recovery protocols, and individual stress responses, influence the relationship between exercise-induced *BDNF* changes and cognitive performance in athletes.

Dopamine, a key neurotransmitter in the brain’s reward system, interacts with *BDNF* to influence motivation, reward-seeking behavior, and stress resilience [13]. Polymorphisms in the *BDNF* gene have been suggested to influence these processes, affecting mood regulation and athletic performance. A common rs6265 (Val66Met; G196A) polymorphism of the *BDNF* gene, which results in a valine (Val) to methionine (Met) substitution, has been linked to reduced hippocampal volume and abnormal hippocampal activation [319,320,321,322]. This Met allele has been associated with smaller hippocampal volumes [323,324] and impaired performance on hippocampus-dependent memory tasks [319], suggesting that reduced BDNF availability may be a risk factor for several psychopathologies, including increased neuroticism, anxiety, depression, and suicide [325,326,327]. These changes in hippocampal structure and function are significant because the hippocampus is involved in mood regulation and memory processing [328,329,330]. Nevertheless, the direct impact of *BDNF* polymorphisms on athletic performance and related personality traits in athletes has not been extensively studied, indicating a critical gap in current sports genetics research.

Our recent study [64] involving 106 martial arts athletes and 152 non-athletic controls showed that MMA athletes with the G/G (Val66Val) genotype of the *BDNF* gene (rs6265) have significantly higher conscientiousness compared to controls and lower neuroticism compared to G/A and A/A genotype athletes, reflecting greater discipline, emotional stability, and resilience to stress. In martial arts, which require discipline and emotional control, these traits are particularly beneficial for focus and stress management during competition. To date, there has been little discussion of how these specific personality traits mediated by *BDNF* polymorphisms contribute to athletic success and performance outcomes. These traits suggest that MMA athletes are more effective in their training and better equipped to handle the pressures of competition, contributing to their overall success in martial arts. These findings are consistent with the study by Asai et al. (2020) [281], who reported a lower prevalence of the Met allele of the *BDNF* Val66Met polymorphism in judo athletes compared to competitive swimmers. This suggests that the Val allele, which is associated with higher BDNF secretion and neuroplasticity, may confer advantages in sports that require rapid adaptation and stress resilience, such as judo. In contrast, the higher prevalence of the heterozygous Val/Met genotype in swimmers suggests potential advantages in motor learning, which is crucial in technically demanding sports, such as swimming. These results highlight the genetic basis of resilience and psychological stress tolerance in different types of sports. Furthermore, another study [292] examined several polymorphisms in the *BDNF* gene, including rs10767664 and rs2030323, and their associations with personality traits in athletes. In a sample of 305 volunteers, including 153 martial arts athletes, the T/T and A/T genotypes of rs10767664 were significantly associated with higher conscientiousness on the NEO-FFI scales. Similarly, the G/G genotype of rs2030323 was associated with higher conscientiousness. However, these associations may be influenced by the interaction between multiple genetic polymorphisms and environmental factors such as training intensity and psychological stressors associated with competitive sports. A possible explanation for the observed associations may be the cumulative effect of multiple genetic factors that collectively influence complex personality traits rather than individual SNP effects. These findings suggest that variations in the *BDNF* gene may influence key personality traits related to athletic performance, although further research is needed to confirm these associations.

### 6.3. Catechol-O-Methyltransferase Gene (COMT) and Personality Traits in Athletes

The *COMT* gene, located on chromosome 22q11.21, encodes catechol-O-methyltransferase, an enzyme crucial for degrading catecholamine neurotransmitters, such as dopamine, norepinephrine, and epinephrine, primarily in the prefrontal cortex [331,332,333]. This brain region plays a vital role in executive functions, including decision-making, emotional regulation, and stress response. A well-documented polymorphism in the *COMT* gene, rs4680, results in a Val158Met (G/A) substitution, which significantly affects COMT enzyme activity [334,335], with Met/Met (A/A) homozygotes having 3–4-fold lower COMT activity than Val/Val (G/G) carriers. This variation leads to increased dopamine levels in the prefrontal cortex [336], affecting critical cognitive functions such as working memory, reward anticipation, and athletic performance [337,338,339,340,341]. The Met polymorphism has also been associated with increased anxiety and unpleasant emotions [335,342] and decreased ability to switch tasks [343]. While these associations are well established, the nuanced ways *COMT* polymorphism influences athletic performance and related personality traits warrant further exploration.

The Val158Met polymorphism in the *COMT* gene shows some relevance in the context of stress tolerance and performance under pressure. Individuals with the Val/Val genotype tend to have higher COMT activity, resulting in faster dopamine degradation and lower baseline dopamine levels in the prefrontal cortex [344]. This genotype is often associated with improved stress resilience and cognitive performance in high-pressure environments [345], which is beneficial for athletes competing in sports that require rapid decision-making under stress, such as soccer, basketball, and tennis. In critical moments, such as penalty kicks or game-winning plays, Val/Val carriers can better maintain focus and emotional stability, which are critical for success in high-stakes competitions [344].

Conversely, athletes with the Met/Met genotype, which results in slower dopamine clearance and higher baseline dopamine levels, may have better baseline cognitive function in low-stress situations. However, they are more susceptible to stress-induced performance declines under high-pressure conditions, making them vulnerable in competitive sporting environments that require rapid cognitive and emotional adjustments [335].

Studies investigating the association between the *COMT* rs4680 polymorphism and athletic performance have yielded mixed results. For example, a study of 57 Asian male competitive swimmers [346] found a positive association between the Met variant and improved performance. In contrast, a study on 225 Polish swimmers, including short-distance and long-distance competitors, found no significant associations between the *COMT* rs4680 polymorphism and athletic performance compared to 379 sedentary controls [347]. Similarly, a study of 60 wrestlers, 55 basketball players, and 60 non-athletes showed no significant differences in the frequency of this polymorphism between elite athletes and controls. These conflicting findings highlight the complex relationship between *COMT* polymorphisms and athletic performance, suggesting that additional moderating factors are involved, such as varying training regimens, environmental stressors, and the study population.

In contrast, our study of 258 combat sports athletes and 278 healthy, non-athletic male controls showed that Val/Val (G/G) carriers in combat sports athletes displayed distinct personality traits that may be beneficial for athletic success [283]. Specifically, athletes with the G/G (Val/Val) genotype showed lower novelty-seeking and higher self-management traits, reflecting better focus, self-control, and stress resilience in high-pressure situations. Additionally, athletes with the G/G genotype showed lower self-transcendence, suggesting a focus on immediate performance goals that might enhance effectiveness in high-pressure situations. Furthermore, this group of combat sports athletes demonstrated lower harm avoidance and higher self-management than controls, highlighting a personality profile that favors risk-taking and discipline, advantageous traits in combat sports. Supporting these findings, Tatar et al. reported a significantly higher frequency of the G/G (Val/Val) “fighter” phenotype in combat sports athletes compared to non-athletes, emphasizing the importance of this genotype in combat sports success [23]. The study highlights the “warrior/worrier” model, in which Met (A) allele carriers, associated with higher dopamine levels, typically outperform Val (G) homozygotes on cognitive tasks under normal conditions. However, under stress, the performance advantage shifts to Val allele carriers, which show better cognitive performance due to more optimal dopamine regulation. The G/G genotype was significantly more common in MMA fighters than in controls (*p* = 0.003), reinforcing that this genotype confers stress resilience and cognitive advantages under pressure, critical traits for success in combat sports. A study of rugby players [287] found that the Val/Val genotype was overrepresented in athletes who had not suffered a concussion, suggesting a protective effect against concussion. In addition, Val/Val participants showed higher “anticipatory worry”, a trait associated with increased readiness and vigilance under pressure. However, there has been limited discussion of how these personality traits, mediated by *COMT* polymorphisms, directly contribute to athletic success and performance outcomes. This is consistent with the “warrior” phenotype, in which Val/Val carriers demonstrate better cognitive resilience and emotional regulation under stress, traits essential for success in high-pressure sports. Further supporting this model, research by Leźnicka et al. analyzing 199 combat sports athletes and 165 non-athletes found that Val/Val homozygous combat athletes exhibited lower sensory sensitivity than Met allele carriers [348]. Moreover, Met/Met homozygotes exhibited lower endurance, whereas Val/Val carriers appeared to be better suited to maintain performance in endurance-demanding combat sports. Such findings are consistent with a study by Stroth et al., where individuals with the G/G genotype of the *COMT* gene experienced more significant improvements in executive control after 17 weeks of aerobic exercise training, as compared to Met carriers [22]. This study involved 75 young adults, assigned to running (*n* = 47) or a control group (*n* = 28). The running group participated in a 17-week running program and demonstrated that increased physical fitness improves cognitive flexibility and executive control, particularly in those with the G/G genotype. These findings suggest that intensive aerobic capacity training may specifically benefit athletes with the G/G genotype, potentially improving their athletic performance through dopaminergic modulation. This is consistent with work by van Breda et al., which demonstrated that Met/Met homozygotes tend to exhibit greater novelty-seeking traits, particularly in endurance sports. This study examined the relationship between the *COMT* Val158Met polymorphism and personality traits in ultra-endurance Ironman athletes (*n* = 55) compared to recreationally active controls (*n* = 32) [295]. The results showed that ultra-endurance athletes scored lower on harm avoidance and psychological distress than controls, suggesting greater emotional resilience and lower anxiety levels. Van Breda et al. also found that Ironman triathletes with the Met/Met genotype expressed higher novelty-seeking and lower harm avoidance, which may increase motivation and adaptability in prolonged events. Notably, Met158 homozygous carriers in the Ironman group had significantly higher novelty-seeking scores, a trait not previously observed in ultra-endurance populations. These findings underscore the potential influence of chronic physical activity on dopamine regulation and personality traits, although the mechanisms underlying these associations remain to be fully elucidated.

Overall, while *COMT* gene polymorphisms such as rs4680 are important in shaping personality traits relevant to athletic performance, the relationship is complex and influenced by multiple factors. There has been limited discussion of how these genetic variations interact with environmental influences and training regimens to optimize athletic performance. Future research should adopt integrative models that consider both genetic and environmental factors to fully understand the role of *COMT*-related genetic polymorphisms in athletic success.

Additionally, most of the studies examining the relationship between dopamine-related genetic polymorphisms and personality traits in athletes have focused on European and North American populations (Table 1). This focus prevents a broader understanding of how these genetic factors may influence athletes from other ethnic backgrounds. Genetic polymorphisms may vary in frequency and effect in different ethnic groups [29,30], potentially leading to different effects on dopamine pathways and associated personality traits. For example, the *ALDH2* rs671 polymorphism is common in East Asian populations and has been associated with both athletic performance and psychological traits such as anxiety and depression [349,350]. The *ALDH2* gene encodes an enzyme involved in alcohol metabolism, and its polymorphisms may indirectly affect dopamine metabolism by modulating oxidative stress and neurotransmitter turnover [351,352].

Studies have shown that interactions between *ALDH2* and *COMT* polymorphisms can influence symptoms of neurological disorders such as Parkinson’s disease [353], suggesting a potential interaction between these genes in dopaminergic pathways. Exploring such interactions in the context of athletic performance may reveal novel genetic factors contributing to personality traits and athletic success in different ethnic groups. This underscores the need to include diverse populations in genetic research to fully understand the complex mechanisms underlying athletic performance.

## 7. Therapeutic Benefits of Physical Activity and Neuroprotection

### 7.1. Exercise as a Neuroprotective Intervention in Addiction Recovery

As reviewed by Lynch et al. and Marrero-Cristobal et al., beyond the immediate benefits, physical activity is also critical for neuroprotection, providing a non-pharmacological intervention for addiction prevention and recovery [354,355]. Physical activity has been increasingly recognized for its role in enhancing brain health and cognitive reserve, which is crucial for resilience against neurodegenerative diseases and addiction [247,354,355,356]. Upregulation of the noradrenergic (NA) system during exercise supports resilience to neurodegeneration and addiction by enhancing catecholamine response. This effect is particularly relevant as recent studies demonstrate that physically trained individuals exhibit a higher VO_2max_—a measure of oxygen uptake and physical fitness—linked to more significant noradrenergic signaling [357]. However, there has been little discussion of the long-term sustainability of these neuroprotective effects and how they may differ across populations and addiction types. This relationship suggests that regular exercise not only aids in addiction recovery, but also bolsters the brain’s resilience to degeneration, potentially offering protective effects against neurodegenerative conditions [357].

Regular physical activity also plays a critical role in regulating dopamine levels in the brain, improving motivation and cognitive function. Exercise has been shown to increase dopamine synthesis, release, and receptor sensitivity [211,358]. Nonetheless, there has been limited investigation into how these dopaminergic changes specifically influence different stages of addiction recovery and relapse prevention. This regulation is essential for maintaining a “feel good” sensation after exercise, often referred to as the “runner’s high”, reinforcing the desire to engage in regular physical activity. Beyond these immediate motivational benefits, physical activity also promotes neuroprotection by enhancing brain health and cognitive reserve, which is critical for resilience to neurodegenerative disease and addiction. Exercise-induced neuroadaptations, such as increased levels of brain-derived neurotrophic factor (BDNF), support synaptic plasticity and neurogenesis, critical for cognitive function and addiction recovery [359,360]. However, there has been little discussion of the optimal types and duration of exercise needed to maximize BDNF-mediated neuroprotective effects in individuals recovering from addiction.

Moreover, aerobic exercise has been shown to have significant effects on the dopaminergic system, particularly in individuals with neurodegenerative diseases such as Parkinson’s disease (PD). A study of PD patients [209] found that regular aerobic exercise increased dopamine release and ventral striatum activity, indicating enhanced dopamine signaling and corticostriatal plasticity. These changes suggest that exercise improves motor function and modulates reward-related brain circuits, potentially aiding in managing both motor and non-motor symptoms of PD. However, there has been little discussion about whether these dopaminergic enhancements translate similarly to individuals recovering from addiction, who may also experience dopaminergic dysregulation. Future research should investigate the parallels and differences in how aerobic exercise affects the dopaminergic systems in these conditions, as another stepping stone towards developing tailored exercise interventions that aim to maximize neuroprotective benefits for each group.

### 7.2. Comprehensive Neuroprotective Benefits of Physical Activity

Physical activity has long been recognized for its broad benefits in promoting physical health, but emerging research underscores its critical role in neuroprotection [356,361]. Regular exercise not only improves cognitive function but also helps delay the onset and progression of neurodegenerative diseases such as Alzheimer’s, Parkinson’s, and multiple sclerosis (MS) [362,363,364,365,366,367,368]. The neuroprotective effects of exercise are largely attributed to its ability to stimulate molecular pathways associated with brain plasticity, cognitive reserve, and stress resilience.

One of the key mechanisms by which physical activity exerts its neuroprotective effects is through the upregulation of Brain-Derived Neurotrophic Factor (*BDNF*), a neurotrophin involved in neurogenesis, synaptic plasticity, and neuronal survival [359,369,370]. Higher levels of BDNF have been associated with improved memory, learning, and overall cognitive performance, particularly in older adults. Regular aerobic exercise has been shown to increase hippocampal volume and BDNF levels, protecting against age-related cognitive decline [370,371].

In addition, exercise contributes to neuroprotection by reducing inflammation and oxidative stress—key contributors to neuronal damage in neurodegenerative diseases. Physical activity reduces pro-inflammatory cytokines, such as TNF-alpha and IL-6, and increases the activity of antioxidant enzymes, thereby limiting the damage caused by reactive oxygen species (ROS) [372,373,374]. These anti-inflammatory and antioxidant effects create a favorable neuronal survival and repair environment. However, it is important to note that these anti-inflammatory and antioxidant benefits of exercise may vary among individuals with different genetic backgrounds and pre-existing health conditions.

The neuroprotective benefits of exercise extend to its role in dopamine regulation, particularly in conditions such as Parkinson’s disease. Exercise has been shown to increase dopamine receptor sensitivity and dopamine release in motor-related brain areas, which may help alleviate motor deficits and improve the quality of life in people with Parkinson’s disease [375,376,377]. In addition, moderate-intensity exercise promotes neuroplasticity, which may slow the progression of dopaminergic neuron degeneration [366].

In the context of mental health, physical activity is increasingly recognized as a non-pharmacological intervention for the prevention and treatment of mood disorders, including depression and anxiety. Exercise-induced changes in neurotransmitters such as serotonin, norepinephrine, and dopamine, along with increased levels of BDNF, contribute to improved mood and emotional resilience [378]. The positive feedback loop between physical activity, enhanced neuroplasticity, and improved mood highlights the potential of exercise as an adjunctive treatment in psychiatric care.

Additionally, genetic predisposition to addiction, such as the presence of the A1 allele of the *DRD2* gene (rs1800497), has been suggested to play an important role in how individuals respond to physical activity. The A1 allele has been consistently associated with addictive behaviors such as alcoholism [379], drug addiction [380], and smoking [381], suggesting that it plays an essential role in the addiction process. Interestingly, the A1 allele was also associated with lower *DRD2* expression [382], which may contribute to an increased risk of addiction due to reduced dopamine receptor availability. However, it has been suggested that exercise may act as a protective factor, especially for individuals carrying the A1 allele [311]. For example, exercise addiction may reduce the tendency to engage in other addictive behaviors, such as smoking or drug use, by increasing dopamine secretion and compensating for the reduction in dopamine receptors, thereby promoting a more balanced dopaminergic response [383,384]. This mechanism underscores the potential of exercise to reduce the appeal of addictive substances. It suggests that genetic variation should be considered in the design of sports participation programs and policies.

Additionally, exercise induces neuroadaptations that may reduce an individual’s risk of initiating drug use. Voluntary exercise increases dopamine receptor binding, particularly at D2 receptors, which are often downregulated in individuals with substance abuse disorders [385,386]. Increased D2R signaling has been associated with greater motivation to overcome addiction, while decreased D2R levels in the striatum have been linked to greater impulsivity and a higher tendency towards addictive behaviors [387]. Studies indicate that moderate, consistent treadmill running can prevent the development of strong conditioned preferences for methamphetamine and inhibit stimulant-induced dopamine increases in critical brain areas such as the nucleus accumbens and striatum [388,389]. These findings confirm that exercise not only provides immediate benefits, but also induces lasting neurobiological changes that diminish the reinforcing effects of drugs, thereby supporting long-term addiction recovery and brain health.

Furthermore, exercise acts as a non-drug reward that competes with addictive substances, reducing their appeal and use. Physical activity induces the mesolimbic reward pathway, which is central to the reinforcing effects of both natural rewards and drugs of abuse [390]. The increase in dopamine release and receptor activation during physical activity is similar to the effects of addictive substances [391,392,393], reducing drug desire and the reward effects of drugs. Acute bouts of exercise boost serum calcium levels, enhancing dopamine synthesis in the brain [394]. Regular exercise further enhances this effect by increasing levels of tyrosine hydroxylase, the enzyme responsible for dopamine production, thereby sustaining higher dopamine levels over time [395,396,397,398].

In addition to its direct effects on the dopaminergic system, physical activity provides numerous secondary health benefits that contribute to overall well-being and addiction recovery. Regular exercise helps prevent obesity and related metabolic disorders, such as diabetes, which are common comorbidities in individuals with substance abuse disorders. By improving cardiovascular health, enhancing metabolic function, and reducing the risk of chronic disease, physical activity provides a holistic approach to addiction treatment beyond drug abstinence alone [399]. Furthermore, recent research suggests that high-intensity interval training (HIIT) can significantly increase levels of selenoprotein P (SEPP1), a biomarker associated with cancer prevention, neurological function, and dopamine signaling. HIIT led to an 84% increase in SEPP1 compared to sedentary controls, suggesting that intense exercise may enhance antioxidant defenses and influence dopamine receptor binding [400]. These findings highlight the potential for personalized exercise therapies to modulate dopamine-related health outcomes and addiction resistance.

Finally, a meta-analysis of 22 independent studies showed that high-intensity exercise improves abstinence rates and reduces withdrawal symptoms [401]. Numerous studies have found that active participation in physical activity reduces the risk of substance abuse and addiction [401,402]. Moreover, exercise has been shown to improve psychological health and cognitive functioning [235,403], further supporting its role in preventing and treating drug dependence. Nevertheless, there has been little discussion about how the combination of high-intensity and other forms of exercise might synergistically enhance neuroprotective and addiction recovery outcomes.

## 8. Conclusions

This narrative review highlights the critical role of dopamine in athletic performance and personality traits, emphasizing the complex interplay between genetic, epigenetic, and environmental factors. The studies reviewed illustrate how variations in dopamine-related genes, such as *DRD2*, *DRD4*, *DAT1*, *BDNF*, and *COMT*, influence an athlete’s predisposition to success through their effects on motivation, cognitive function, and emotional resilience. These genetic variations are not isolated; they dynamically interact with environmental factors such as training intensity, stress, and recovery and modulate dopamine pathways through mechanisms such as DNA methylation.

Emerging research in this area underscores the potential for personalized athletic training strategies based on an individual’s genetic and epigenetic profile. By understanding how these biological factors shape personality traits and athletic ability, coaches, sports psychologists, and athletes can develop more targeted training and performance optimization approaches. This could lead to tailored interventions that enhance physical performance, mental toughness, and psychological well-being, ultimately contributing to an athlete’s overall success and longevity in their sport.

It is clear, however, that much remains to be discovered in this burgeoning area of sports science. The study of personality traits in athletes, particularly as they relate to genetic and epigenetic factors, is still in its infancy. Future research should deepen our understanding of how these traits develop and interact with biological and environmental factors. Such efforts will advance our knowledge of the molecular basis of athletic performance and pave the way for more effective, individualized training programs that capitalize on an athlete’s unique biological strengths and address potential weaknesses.

Additionally, future research should aim to include more diverse populations in genetic studies examining personality traits and athletic performance. The current focus on European and North American athletes may not capture the full spectrum of genetic diversity, potentially overlooking important polymorphisms that influence dopamine pathways and associated traits in other populations.

Investigating interactions between ethnicity-specific genes and dopamine-related genes may reveal novel mechanisms affecting neurological function and personality traits relevant to athletic success. By expanding the diversity of study populations, researchers can develop a more comprehensive understanding of the genetic and epigenetic factors influencing athletic performance, ultimately leading to more personalized and effective training and intervention strategies for athletes worldwide.

While the mechanisms linking dopamine, genetics, epigenetics, and athletic performance are complex and not yet fully understood, the evidence points to a promising frontier in sports science. As we continue to unravel these intricate relationships, the potential to enhance athletic performance through personalized approaches becomes more tangible, leading to a new era of optimized training, enhanced recovery, and mental health strategies that are tailored to the needs of individual athletes.

In conclusion, recognizing the polygenic and multifactorial nature of athletic performance is essential for advancing personalized sports medicine and athlete management. Emphasizing polygenic profiles and SNP–SNP interactions provides a more comprehensive understanding of how genetic factors influence both physical and psychological aspects of athletic performance. Future research should focus on integrating these complex interactions into predictive models and training interventions to optimize performance and promote overall athlete well-being.

## Figures and Tables

**Table 1:** Genetic polymorphisms and their association with personality traits in athletes.

Study	Gene: Polymorphism	Participants	Results
Abrahams et al., 2019 [280]	*DRD2:* rs12364283 (A/G), rs1076560 (C/A) *DRD4:* rs1800955 (T/C) *DRD2:* rs12364283 (A/G), rs1076560 (C/A)	Junior rugby players (*n* = 135) Club and professional rugby players (*n* = 166)	The rs1800955 TT and CT genotypes were associated with low reward dependence in juniors (*p* < 0.001) and seniors (*p* = 0.010), respectively. The rs1800955 C/C genotype was associated with decreased concussion susceptibility in juniors, while the *DRD2*-*DRD4* A-C-C allele combination was also linked to reduced susceptibility. High harm avoidance lowered concussion risk in juniors (*p* = 0.009) but increased it in seniors (*p* = 0.001).
Asai et al., 2020 [281]	*BDNF:* rs6265 (G196A; Val66Met)	Male competitive swimmers (*n* = 105) Male judo athletes (*n* = 74) Male healthy non-athletes (*n* = 87)	Lower prevalence of the Met allele in judo athletes compared to competitive swimmers. Higher frequency of heterozygous Val/Met *BDNF* polymorphism in competitive swimmers.
Chmielowiec et al., 2021 [282]	*DRD2*: rs1799732 (C/-)	Polish male MMA (*n* = 258) Polish male controls (*n* = 284)	Deletion variant associated with lower reward dependence and harm avoidance in MMA athletes.
Humińska-Lisowska et al., 2022 [64]	*BDNF*: rs6265 (G196A; Val66Met)	Polish martial arts athletes (*n* = 106) Polish controls (*n* = 152)	G/G genotype (Val/Val) associated with higher conscientiousness and lower neuroticism.
Humińska-Lisowska et al., 2023 [283]	*COMT*: rs4680 (Val158Met; G/A)	Polish male combat sports athletes (*n* = 258) Polish male controls (*n* = 278)	G/G genotype (Val/Val) associated with lower novelty-seeking, lower self-transcendence, and higher self-management scores in combat sports athletes.
Humińska-Lisowska et al., 2024 [284]	*HTR1A*: rs6295 (C-1019G)	Polish male MMA (*n* = 250) Polish male controls (*n* = 209)	G/G genotype associated with higher self-management and lower harm-avoidance scores in combat sports athletes.
Huminska-Lisowska et al., 2024 [285]	*DRD2*: Taq1D rs1800498 (C/T)	Polish athletes (*n* = 159) Polish controls (*n* = 232)	Associated with higher conscientiousness in athletes.
McAuley et al., 2022 [286]	*HTR2A*: rs6311 (C/T) *BDNF*: rs6265 (C/T; Val/Met) *CHRM2*: rs1824024 (C/A) *COMT*: rs4680 (G/A; Val/Met) *CTNNA2*: rs7600563 (T/G) *DRD2*: rs1800497 (G/A; Glu/Lys) *DRD3*: rs167771 (G/A) *DRD4*: rs1800955 (T/C) *GABRA6*: rs3219151 (C/T) *OXTR*: rs2254295 (C/T)	English male football players (*n* = 73)	The A/A genotype of *DRD3* (rs167771) was associated with higher levels of agreeableness compared to the G allele.
Mc Fie et al., 2017 [287]	*COMT*: rs4680 (Val158Met; G/A) *SLC6A4*: 5-HTTLPR rs25531	Case male rugby players from RPA (*n* = 163) Control male rugby players from RPA (*n* = 140)	*COMT* Val/Val: More common in non-concussed rugby players, linked to increased anticipatory worry in juniors. Low 5-HTTLPR: Less frequent in non-concussed; juniors showed reduced harm avoidance, anticipatory worry, and fear of uncertainty.
Michałowska-Sawczyn et al., 2019 [288]	*DRD4* Ex3 VNTR	Polish male combat sports athletes (*n* = 151) Polish male controls (*n* = 130)	Ex3 l/l genotype associated with higher conscientiousness in CS. Ex3 s/s genotype associated with lower openness in CS.
Michałowska-Sawczyn et al., 2020 [289]	*DAT1* 3′ UTR VNTR polymorphism	Polish male combat sports athletes (*n* = 200) Polish male controls (*n* = 102)	9/10 VNTR genotype associated with lower levels of anxiety in CS. 10/10 VNTR genotype associated with lower agreeability in CS.
Michałowska-Sawczyn et al., 2025 [290]	*DRD2:* rs1079597	Polish professional athletes (*n* = 163) Polish controls (*n* = 232)	G/G genotype and G allele were more frequent in athletes. Athletes with G/G, A/G, and A/A genotypes had higher conscientiousness compared to controls. G/G athletes showed lower openness than those with the A/G genotype, while A/G athletes displayed higher openness compared to controls across all genotypes.
Niewczas et al., 2021 [291]	*DRD2*: rs1799732 (C/-)	Polish MMA (*n* = 85) Polish controls (*n* = 284)	Deletion variant associated with lower reward dependence in MMA athletes.
Niewczas et al., 2021 [292]	*BDNF*: rs10767664 (A/T), rs2030323 (T/G)	Polish MMA (*n* = 151) Polish controls (*n* = 130)	T/T and A/T genotypes of rs10767664 and G/G genotype of rs2030323 significantly associated with higher conscientiousness in MMA compared to controls.
Stroth et al., 2010 [22]	*COMT*: rs4680 (Val158Met; G/A)	Runners (*n* = 47) Control group (*n* = 28)	G/G (Val/Val) runners showed greater cognitive gains than Met carriers after 17 weeks of aerobic training.
Tartar et al., 2020 [23]	*COMT*: rs4680 (Val158Met; G/A)	Male participants of mixed ethnicities: - MMA = 21 - athletes = 21 - non-athletes = 41	Val allele linked to enhanced stress resilience (“warrior/worrier” model) in MMA.
Thomson et al., 2013 [293]	*DRD4*: rs180095 (−521 C/T)	Skiers and snowboarders (M = 287; F = 216); Caucasian	Higher sensation-seeking (novelty-seeking) scores in the −521 C/C genotype group.
Thomson et al., 2013 [294]	*COMT*: rs737865, rs4633, rs4680, rs165599 *SLC6A3* or *DAT1*: rs6347, rs27072, rs463379, rs2937639, rs2975226 *DBH*: rs1611115 *DRD1*: rs686, rs4532, rs251937, rs4867798 *DRD2*: rs6277, rs1076560, rs1079597, rs1800497, rs1800498, rs2283265, rs2734831, rs4245147, rs7131056, rs17601612 *DRD3*: rs6280, rs167771 *MAO*-*B*: rs1799836 *HTR2A*: rs6311	Skiers and snowboarders (M = 341, F = 258); Caucasian	Lower sensation-seeking scores were associated with the G allele of the *DRD3* gene (rs167771).
van Breda et al., 2015 [295]	*COMT*: rs4680 (Val158Met; G/A)	Ultra-endurance Ironman athletes (*n* = 55) Recreationally active controls (*n* = 32)	A/A (Met/Met) homozygotes in Ironman athletes showed higher novelty-seeking, lower harm avoidance and psychological distress.

*BDNF*—brain-derived neurotrophic factor; *CHRM2*—Cholinergic Receptor Muscarinic 2; *COMT*—catechol-O-methyltransferase; *CTNNA2*—Catenin Alpha 2; *DBH*—Dopamine Beta-Hydroxylase; *DRD*—dopamine receptor D; *GABRA6*—Gamma-Aminobutyric acid type A Receptor subunit Alpha6; *HTR1A*—5-hydroxytryptamine receptor 1A; *HTR2A*—5-hydroxytryptamine receptor 2A; 5-HTTLPR—serotonin-transporter-linked polymorphic region; *MAO-B*—monoamine oxidase B; *OXTR*—oxytocin receptor; *SLC6A4*—Solute Carrier family 6 member 4; CS—combat sports; F—female; M—male; MMA—mixed martial arts; VNTR—variable number tandem repeat.

## Data Availability

The original contributions presented in this study are included in the article. Further inquiries can be directed to the corresponding author.
